# Mgat4b-mediated selective *N*-glycosylation regulates melanocyte development and melanoma progression

**DOI:** 10.1073/pnas.2423831122

**Published:** 2025-05-27

**Authors:** Babita Sharma, Keerthic Aswin, Tanya Jain, Ayesha Nasreen, Ayush Aggarwal, Yogaspoorthi J. Subramaniam, Jeyashri Rengaraju, Srashti Jyoti Agrawal, Mayank Bhatt, Bhashkar Paul, Koushika Chandrasekaran, Aanchal Yadav, Jyoti Soni, Rajat Ujjainiya, Md Quasid Akhter, Rajesh Pandey, Shruthy Suresh, Srinivasa-Gopalan Sampathkumar, Vivek T. Natarajan

**Affiliations:** ^a^Council of Scientific and Industrial Research-Institute of Genomics and Integrative Biology, New Delhi 110025, India; ^b^Academy of Scientific and Innovative Research, Ghaziabad, Uttar Pradesh 201002, India; ^c^Laboratory of Chemical Glycobiology, National Institute of Immunology, New Delhi 110067, India

**Keywords:** melanocyte, N-glycosylation, cell migration, cell adhesion, melanoma

## Abstract

Melanocyte development involves key pathways that are often recapitulated during melanoma initiation, highlighting the importance of understanding these regulatory processes. Our study identifies *mgat4b*, a glycosyl transferase involved in selective *N*-glycan branching enriched in pigment progenitors, as a regulator of directional cell migration and establishment of melanocyte stem cell (McSC) pool during early development. Single cell RNA (scRNA) sequencing analysis in zebrafish upon targeted disruption of *mgat4b* reveals, that migratory melanocyte progenitors marked by galectin expression fail to persist. Lectin affinity proteomic analysis reveals the glycosylation of key melanocyte proteins GPNMB, KIT, and TYRP1 to be under the control of MGAT4B in melanocytic cells. Additionally, mislocalization of Junctional plakoglobin (JUP) explains the observed defects in cell adhesion and migration to be regulated by MGAT4B but not its isozyme MGAT4A. Our meta-analysis further reveals that melanoma patients with both the BRAF^V600E^ mutation and elevated MGAT4B levels have significantly worse survival outcomes compared to those with only the BRAF^V600E^ mutation. By leveraging the zebrafish MAZERATI platform to model BRAF^V600E^ driver mutation in vivo, we show that *mgat4b* mutant cells fail to aggregate and initiate tumors. RNA profiling of the transformed melanocytes revealed cell–cell junction, adhesion, and ECM binding to be probable contributing factors that resulted in the failure of tumor onset. Using a small-molecule inhibitor we demonstrate that complex *N*-glycosylation inhibits early-stage melanoma progression. Our study underscores the importance of selective *N*-glycan branching in both melanocyte development and melanoma initiation, suggesting MGAT4B as a promising therapeutic target for melanoma treatment.

## Significance

This study reveals that the enzyme MGAT4B, which adds sugar molecules to proteins, plays a key role in how pigment-producing skin melanocytes move and organize themselves. This enzyme controls key melanocytic proteins involved in movement and adhesion. This is necessary for pigment progenitor to establish themselves correctly, and blocking it can prevent progression of early melanoma. Our findings challenge the idea that protein sugar-tagging is the same in all cells and show how developmental pathways are reused in cancer. These findings offer a promising therapeutic target, paving the way for innovative, targeted melanoma treatments.

Melanocytes are specialized cells that originate from neural crest cells (NCCs) and contain melanin-rich melanosomes. These cells are crucial for skin pigmentation that serves a protective function against DNA damage (1). Early specifying melanocytes directly originate from the neural crest and migrate over the developing vertebrate embryo to orchestrate the pigmentation pattern (2, 3). During regeneration, these cells are replenished by tissue resident melanocyte stem cell (McSC) pool (4). Unraveling the molecular mechanisms of melanocyte development is vital not only for understanding early development but also for combating diseases like melanoma, as these developmental processes are often co-opted during the transformation of melanocytes to melanoma and its further progression (5, 6).

Recent research has elucidated important pathways such as Kit and ErbB signaling involved in establishing McSCs (4, 7–9). Melanocytes originate directly from NCCs and from multipotent progenitor cells that reside in tissues but are derived from NCCs during early development (3). The transcription factor Mitfa plays a central role in this process, with additional support from factors like Tfap2a-c (10). Thyroid hormone influences terminal differentiation, limiting the final numbers of mature melanocytes (11). Adult pigment cell stripes are formed by the Schwann cell precursors providing a secondary source of melanocytes, marked by a distinct gene expression pattern (12–14). Furthermore, recent studies reveal an important role for the Prl3-Ddx21 axis, in preserving McSC pools (15). Interestingly these factors also have a central role to play in melanoma cells, highlighting the recapitulation of developmental programs in melanoma (15–18).

There is growing evidence that epigenetic and transcriptional developmental programs specific to melanocytes and their precursors are reexpressed during melanoma initiation (5, 6). It is important to identify the programs regulating melanocyte cell states as this may help us identify and target early events in melanoma initiation. In this study, we set out to identify factors that promote melanocyte differentiation from NCCs and found that a glycosyl transferase *mgat4b* initiates the formation of a unique *N*-glycan branch on set of glycoproteins, and functions as a crucial orchestrator of melanocyte development. This finding is intriguing, as recent studies highlight the role of metabolic pathways and glycans in driving embryo growth, along with cellular signaling systems that regulate cell fate decisions and cellular movement during early development (19).

We report *mgat4b*, a selective *N*-glycosylation enzyme, is enriched in pigment progenitors and melanophores, where it regulates melanocyte migration and patterning in zebrafish and mammalian cells. Its loss disrupts Galectin1 marked migratory cells and alters key glycoproteins (GPNMB, KIT, TYRP1) and adhesion protein JUP. MGAT4B is overexpressed in human melanoma and is essential for melanoma initiation, as shown using the MAZERATI in vivo platform (20). Thus, our work highlights the role of selective *N*-glycosylation in early-stage melanocyte development and how this impacts melanoma initiation. These findings underscore the importance of selective *N*-glycan branching in melanoma development and positions MGAT4B as a promising target for melanoma therapy.

## Results

### *mgat4b* Is Enriched in Pigment Progenitors and Melanophores.

**Fig. 1.**
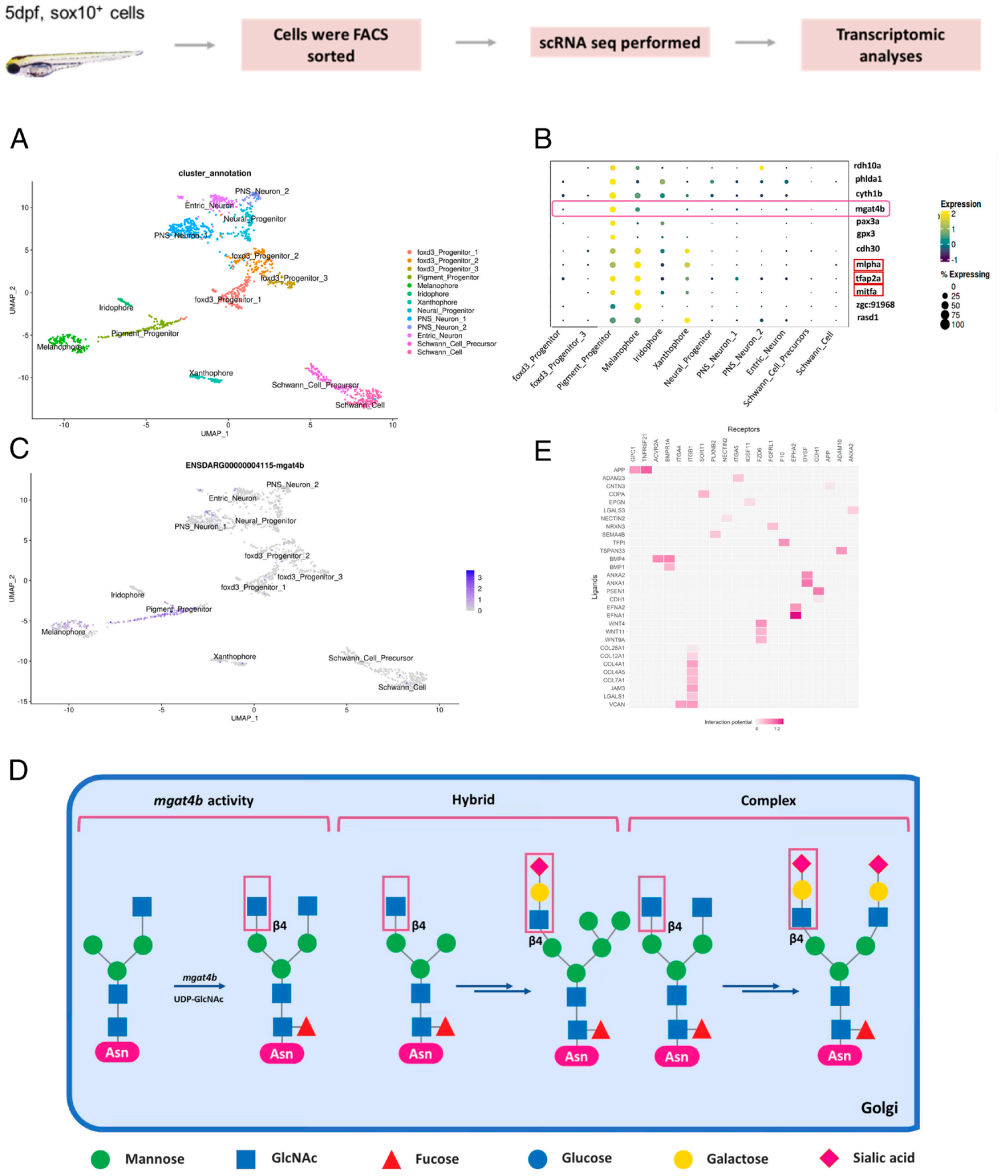
*mgat4b* is enriched in pigment progenitors and melanophores. (*A*) UMAP visualization of the Zebrafish Sox10+ve cells colored by the identified states. (*B*) Dot plot showing the top marker genes enriched in each cluster with the size showing the percent of cell expressing the gene and color showing the scaled mean expression value in each cluster (Wilcoxon–Mann–Whitney test with average log fold change > 0.25 and adjusted *P* value ≤ 0.05). List includes known cell-type marker genes (marked in red box) and new candidate markers. (*C*) UMAP plot shows enrichment of *mgat4b* in pigment progenitor and melanophore clusters. (*D*) Mammalian protein *N*-glycosylation starts in the endoplasmic reticulum and continues in the Golgi apparatus, where three mature types of *N*-glycan structures—high-mannose, hybrid, and complex—are formed. The enzyme MGAT4B transfers *N*-acetylglucosamine in a β1 → 4 linkage to α1 → 3-linked mannose, which initiates the creation of a specific *N*-glycan branch. This branch often includes a sialylated *N*-acetyllactosamine sequence, highlighting its distinctive features in glycan structure. (*E*) Heatmap of NicheNetR-identified ligand/receptor pairs indicating interaction potential between *mgat4b*+ve receiver cells and other sender cells sampled by scRNAseq of zebrafish Sox10+ve cells.

In an anticipation of finding factors that favors melanocyte development from NCCs, we performed reanalysis of zebrafish scRNA data in NCCs (11). Dimensionality reduction and unsupervised clustering was applied to the entire population of sox10+ cells, leading to the identification of markers specific to pigment progenitors and developing melanocytes (Fig. 1*A*). As expected, *mitfa*, *tfap2a,* and *mlpha* were enriched in melanophore and pigment progenitors (Fig. 1*B*). Interestingly, we observed that *mgat4b*, a gene encoding a glycosyl transferase, was preferentially enriched in melanophores compared to other chromatophores which arise from the same cell population of pigment progenitors (Fig. 1*B*). Mgat4b expression was higher in the progenitors compared to mature melanophores, suggesting an early role, if any (Fig. 1*C*). Mgat4b is an enzyme located in Golgi-apparatus that forms β1 → 4 linked branch on α1 → 3 mannose residues of selective *N-*glycoproteins (Fig. 1*D*). In this subpopulation of *mgat4b*-expressing cells, receptor–ligand analysis highlighted key factors involved in cell migration, such as Plexin b2 and Nectin2, which are crucial for migration-related signaling. Additionally, the presence of Integrin α4, α5, and α1, along with Adam10, is suggestive of their role in interactions with the extracellular matrix essential for effective migration (Fig. 1*E*) (21). Since many of the predicted targets of *mgat4b* modulate cell surface receptor signaling and govern cell–cell or cell–matrix adhesion, we reasoned that *mgat4b* may play a critical role in melanocyte patterning and development.

### Global Targeting of *mgat4b* Results in Melanophore Patterning Defects.

To understand the role of *mgat4b* in melanocyte development, we adopted three independent strategies. First, we used a splice block morpholino against *mgat4b* to transiently knockdown the gene. As a result, we observed a significant reduction in Mgat4b protein levels using Western blotting (*SI Appendix*, Fig. S1). Further we observed mild head and eye size reduction in the morphants (*SI Appendix*, Fig. S1 *A* and *B*). We analyzed these animals till day 5, when the melanophores are well patterned, melanized, and all five melanophore stripes dorsal, ventral, yolk sac, and two lateral are visible. The *mgat4b* morphants showed a significantly lower number of lateral mid-line melanophores with respect to control animals (*SI Appendix*, Fig. S1 *C* and *D*).

To assess the initial melanophore emergence along the crest, we utilized a transgenic zebrafish line Tg(*mitfa*:GFP). Fluorescence imaging conducted at 24 h post fertilization (hpf) showed reduction in emergent streams of *mitfa*+cells from the crest in the absence of *mgat4b* (*SI Appendix*, Fig. S1 *E* and *F*). Furthermore, using a Tg(f*tyrp*1: GFP) line where GFP expression is specific to differentiating melanophores, we found aberrant melanophores patterning in the trunk region upon *mgat4b* knockdown at 2 d postfertilization (dpf) (*SI Appendix*, Fig. S1 *G* and *H*).

Second, to complement the morpholino approach, we generated CRISPR mediated global knockout of *mgat4b in vivo* by injecting the sgRNA-Cas9 RNP complex at the single-cell stage (*SI Appendix*, Fig. S2*A*). Brightfield imaging of F0 animals and nontargeting control embryos at 3dpf revealed patterning defects, with a notable portion of melanophores becoming arrested along the path (*SI Appendix*, Fig. S2 *A* and *B*). Sequencing confirmed in-del mutations disrupting the coding sequence of *mgat4b* in F1 adult fishes (*SI Appendix*, Fig. S2*C*). These sequence-validated F1 animals were in-crossed to obtain the F2 generation. Approximately 75% of the F2 embryos displayed a reduced number of lateral melanophores compared to wild-type animals (*SI Appendix*, Fig. S2 *D* and *E*). We assessed the zygosity status of these F2 embryos by performing High Resolution Melt Analysis (HRMA) on phenotypically positive animals and raised them to adulthood. The resulting F2 adults also showed a reduction in adult melanophore numbers (*SI Appendix*, Fig. S2 *F* and *G*). Thus, the global knockout of *mgat4b* (*mgat4b* KO) recapitulated the morpholino phenotype, with a decrease in the number of lateral melanophores and overall patterning defects.

### Melanocyte Specific Ablation of *mgat4b* Leads to Patterning Abnormalities.

Third, in order to assess whether the defects observed were due to melanocyte specific functions of *mgat4b*, we adopted a cell-type specific mutation strategy by modifying the MinicoopR-sgRNA construct developed for rescuing melanophores in casper animals (20). We replaced *mitfa* ORF with a visualization marker GFP, so that melanocyte specific expression of this construct could be visualized through coexpression of GFP with a concomitant ability to target individual genes in melanophores (*SI Appendix*, Fig. S3*A*). We injected this *mitfa*: Cas9, *mitfa*: gfp plasmid at the single cell stage and observed majority of melanophores labeled at 2dpf (*SI Appendix*, Fig. S3*A*). This construct was specifically expressed in melanocytes as GFP+ sorted cells showed enrichment for melanocyte-specific markers such as *mitfa, dct,* and *tyr* at 2 dpf (*SI Appendix*, Fig. S3*B*). Whole mount in-situ (WISH) was used to check for Cas9 RNA expression, which was well restricted to areas where melanophores reside based on the position of the GFP signal (*SI Appendix*, Fig. S3*C*) (22).

We cloned *mgat4b* sgRNAs into this *mitfa*:Cas9;*mitfa*:gfp plasmid construct to specifically ablate *mgat4b* in melanophores. The efficiency of sgRNAs was evaluated using a T7 endonuclease assay on sorted GFP+ cells from both samples. Using qRT-PCR we observed a significant reduction in *mgat4*b transcript levels within melanocytes isolated by FACS (*SI Appendix*, Fig. S3 *E* and *F*). Targeting *mgat4b* specifically within melanophores (*mgat4b* mut) revealed patterning defects at the 3 dpf stage. By 5 dpf, there was a notable decrease in the number of lateral melanophores, (*SI Appendix*, Fig. S3 *G*–*J*). Thus, all three experimental approaches that were employed to target *mgat4b* consistently showed a reduction in the number of embryonic lateral-line melanophores. Thereby, our somatic cell-type ablation strategy highlights that gene ablation in melanocyte specific manner in vivo is a powerful tool for screening candidates and evaluating their roles using pigmentation outcomes as a readout. This strategy highlights the melanocyte-specific role of *mgat4b*, demonstrated by the mutant’s inability to form lateral melanophores. To unravel the mechanistic basis of this phenotype, we utilized single-cell RNA sequencing in GFP-positive cells.

### scRNA Data Map the Fate of *mgat4b* Targeted Melanocyte Precursors.

**Fig. 2.**
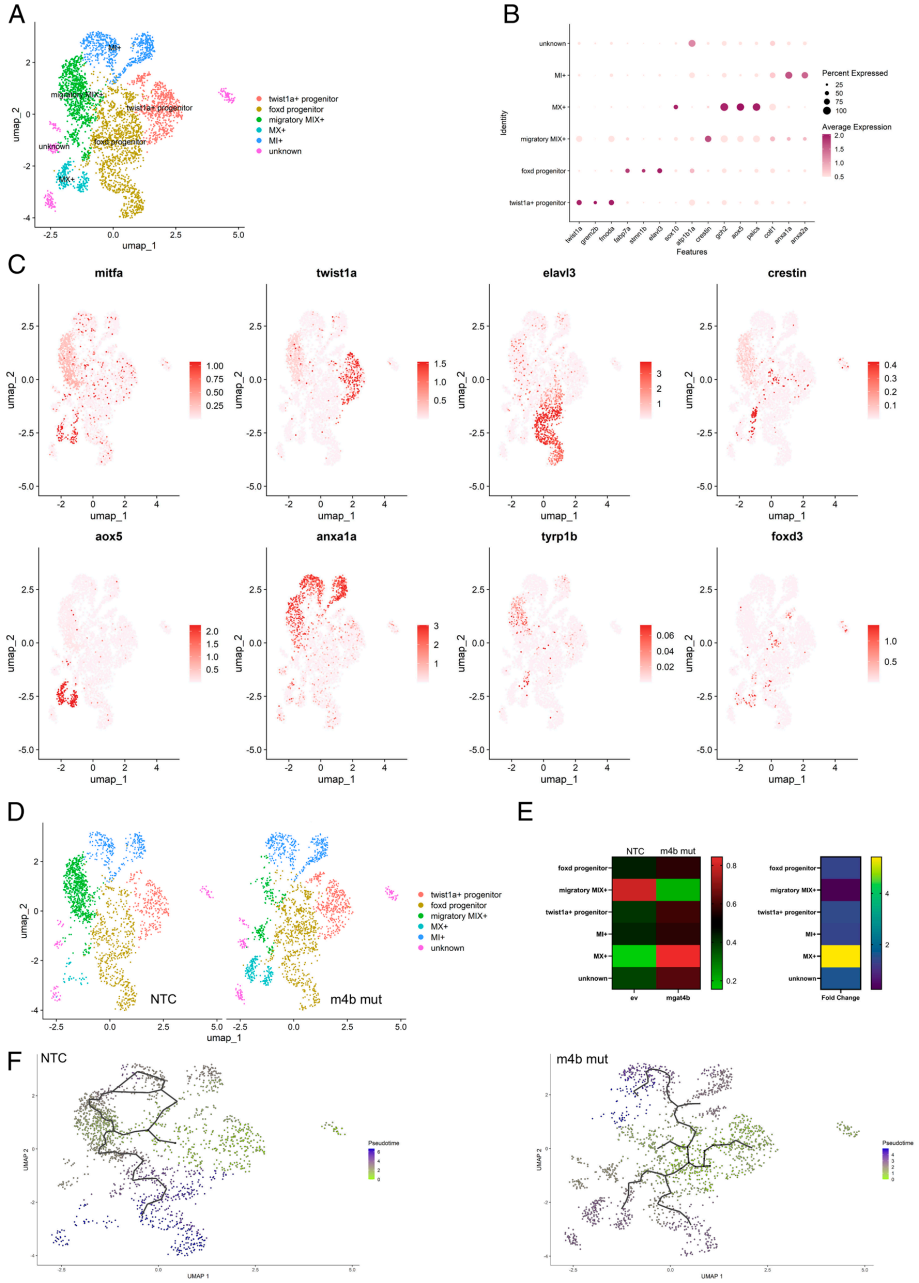
Melanocyte specific ablation of *mgat4b* leads to cell-arrest and results in cell death. (*A*) UMAP visualization of the zebrafish *mitfa*+ve cells integrated from nontargeted control (NTC) and melanocyte specific knock out of *mgat4b* (m4b mut)cells colored by the identified states. (*B*) Dot plot showing the top marker genes enriched in each cluster with the size showing the percent of cell expressing the gene and color showing the scaled mean expression value in each cluster. (*C*) UMAPs of *mitfa*+ cells with color change from light pink (negative) to red based on log normalized scaled expression of *mitfa, twist1a, elavl3, crestin, aox5, anxa1a and tyrp1b* and *foxd3*. (*D*) UMAP visualization of the Zebrafish *mitfa*+ve cells from NTC and *mgat4b* mut cells colored by the identified states *E* (*Left* panel). Heat map showing the proportion of cells belonging to certain cell-type distributed between NTC and *mgat4b* mut samples *E* (*Right* panel). Heat map depicting fold change of the cell number across NTC and *mgat4b* mutant sample. (*F*) Pseudotime ordering of the cells in NTC (*Left*) and *mgat4b* mutant (*Right*), coloring based on pseudotime scores (Wilcoxon–Mann–Whitney test with average log fold change > 1 and adjusted *P* value ≤ 0.05).

Our data thus far demonstrated that *mgat4b* mutant results in dysregulation of melanophore development. However, *mgat4b* loss seems to specifically impact lateral melanophores more than the dorso-ventral ones. Thus, to identify the key melanophore populations altered by *mgat4b*, we performed single-cell RNA sequencing (scRNA-seq) on Nontargeting controls (NTC) and somatic melanocyte-specific *mgat4b* mutant (*mgat4b* mut) GFP+ cells at 36 hpf using the 10× Chromium platform. After integrating all samples, we identified six distinct clusters (Fig. 2 *A* and *B*). Based on markers from previously published datasets (4, 11), we assigned the identities for these clusters (Dataset S5). We identified two proliferative neural crest (NC) populations: *twist1a*+ NC progenitors and *foxd*+ progenitors (Fig. 2*C*). Additionally, we identified three distinct clusters expressing combination of different chromatophore markers. One cluster expressed markers of all three types of chromatophores namely xanthophore, melanophore, and iridophore (expressing *aox5, mitfa, pnp4a*), stem cell markers (*crestin, sox10*), and migratory markers (*rac2, csf3b, rgs2, and itga6*) (Fig. 2*C* and *SI Appendix*, Fig. S4*D*). Hence, we designated this cluster as the migratory MIX+ (migratory melanophore, iridophore, and xanthophore) population. We observed this cluster to be also enriched for *tyrp1b* and not *foxd3* (Fig. 2*C*), therefore, it is likely that they will eventually become mature melanophores (23). In addition, we identified two bipotent clusters, MX+ (melanophore-xanthophore) and MI+(melanophore-iridophore) (Dataset S5). Representative markers for each cluster are shown in the dot plot and UMAP (Fig. 2 *B* and *C*) and top ten markers of each cluster are shown as heatmap (*SI Appendix*, Fig. S4*E*). Importantly, the migratory MIX+ cluster was specifically depleted in the *mgat4b* mut group suggesting that these cells may contribute to the melanocyte defects observed in vivo (Fig. 2 *D* and *E*). Further analysis revealed that lectin genes such as *lgals1, lgals3, and lgals9* are highly enriched in the MIX+ cluster (*SI Appendix*, Fig. S4*F*). These lectin genes have galactoside-binding properties, and are known to interact with substrates that contain branched structures modified by Mgat4 (24). This suggests a critical requirement of specific *N*-glycosylation branching mediated by *mgat4b* for the sustenance of this migratory population.

Functional enrichment analysis of genes in the missing cluster identified significant enrichment of ECM binding, cell adhesion, and galectin binding (*SI Appendix*, Fig. S4*C*). We then performed cell–cell communication analysis to understand the cellular interactions in this cluster (*SI Appendix*, Fig. S4 *A* and *B*). The results confirmed our earlier observation of their migratory potential, as these cells expressed key receptors and metalloproteases (*itga6, rac2, mmp9, cxcl14,* and *mmp*30) essential for cell migration, adhesion, and chemotaxis-related processes (*SI Appendix*, Fig. S4*F*). To investigate the lineage relationships between clusters, we conducted a pseudotime analysis based on gene expression that revealed a trajectory. A branch point originating from NCs that transitioned into MX+ and MI+ through migratory MIX+ cells (*SI Appendix*, Fig. S4*D*). The specific reduction in the MIX+ cluster caused by the *mgat4b* mutation suggests an effect on melanoblast formation, and led to noticeable relative increase in MX+ and MI+ cell numbers (Fig. 2 *D* and *E*). To investigate whether the fate of MIX+ is changed to MX+ or MI+ in the absence of *mgat4b*, we created separate pseudotime trajectories for both the samples and observed that the fate of early *foxd3*+progenitor population shifts toward the MX+ cluster rather than the MIX+ cluster, as seen in the control sample (Fig. 2*F*). This finding suggests that loss of *mgat4b* drives early neural crest progenitors to transition directly into MX+ or MI+ lineages, bypassing the MIX+ state. These are likely to differentiate into xanthophores or iridophores, as indicated by the expression of markers like *aox5* for xanthophores and *anxa1a* and *anxa2a* for iridophores (Fig. 2*C*). Consistent with this, there is no reduction in the xanthophore and iridophore populations in the *mgat4b* mut group. To summarize, through single cell transcriptomics, we identified that selective *N*-glycan branching mediated by *mgat4b* in migratory melanophore precursors is critical and essential for the early migration, directionality, and survival of cells that develop into melanophores.

### Mgat4b Targeting Leads to Migration Arrest and Subsequent Cell Death.

**Fig. 3.**
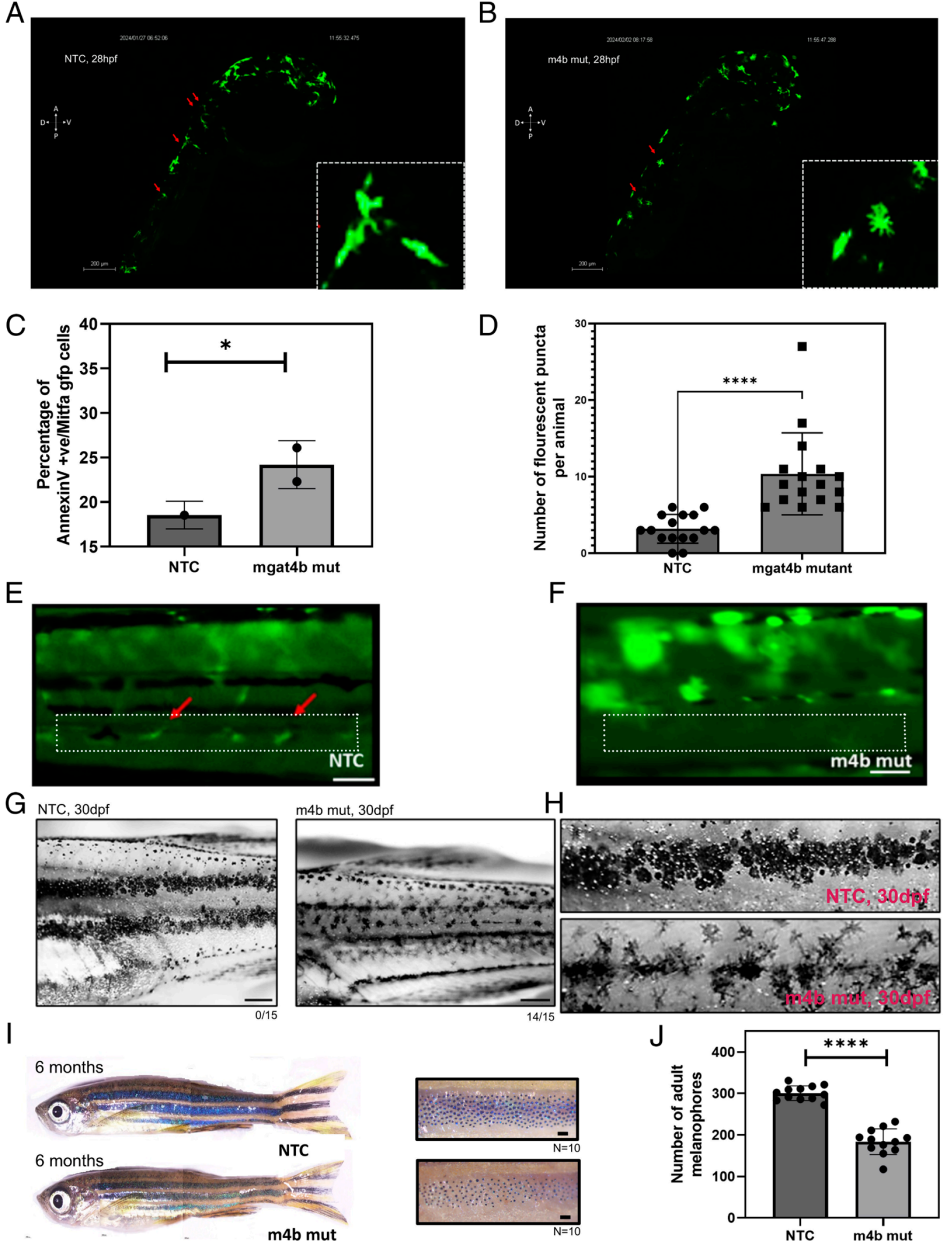
Melanocyte specific ablation of *mgat4b* leads patterning abnormalities and reduced adult melanophores. (*A* and *B*) Snapshot of time-lapse imaging showing early melanophore migration. Red arrows in NTC animal shows elongated melanophores migrating in dorsolateral or dorsomedial direction (*Inset*) whereas in *mgat4b* mut animal they mark direction-less star-shaped melanophores (*Inset*). (Scale bar, 200 µm.) (*C*) The bar graph represents mean ± SEM of percentage of Annexin V+/*mitfa* gfp+ cells in NTC and *mgat4b* mutant animals, n = 3, unpaired t test, *P* = 0.0138. (*D*) The bar graph represents mean ± SEM of number of fluorescent puncta per animal in NTC and *mgat4b* mutants, n = 2, unpaired *t* test, *P* ≤ 0.0001. (*E* and *F*) Lateral view of tissue specific NTC and *mgat4b* mut at 7dpf showing melanocyte stem cells (McSCs) residing pattern. The red arrows and the white dotted box highlight the distribution of McSCs in the trunk region. (*G* and *H*) Lateral view of 1 mo old NTC and *mgat4b* mut animal showing pigment stripes after metamorphosis (*Left*) zoomed images showing well, melanized irregularly circular shaped melanophores in NTC and dendritic star shaped melanophores in mutant (*Right*) 14/15 indicates 14 out of 15 fish imaged as shown in the representative image show the aberrant phenotype. (Scale bar, 50 µm.) (*I*) Lateral view of 6 mo old NTC and *mgat4b* mut animal showing adult pigment stripes, the zoomed images on the right shows constricted melanophores upon epinephrine treatment. (Scale bar, 1,000 µm.) (*J*) The bar graph represents mean ± SEM of melanophore count in adult stripes in NTC and *mgat4b* mut animals *P ≤ 0.05, **P ≤ 0.01, ***P ≤ 0.001, ****P ≤ 0.0001 and ns *P* > 0.05.

The NCC derived progenitors migrate dorso-medially to establish the McSC pool (7). We therefore traced the dorso-medial migration of NCC derived progenitors. Time-lapse imaging of NTC and *mgat4b* mut animals showed directional migration of melanophores, with elongated cells extending in the direction of movement (Fig. 3*A*, *Inset* and Movie 1). In contrast, the *mgat4b* mut animal exhibited melanophores extending dendrites in all directions Fig. 3*B*, *Inset*. These melanophores appeared to be arrested along their paths and showed very limited movement (Fig. 3*B* and Movie 2). We analyzed the velocity and directionality of these cells as they migrated from the neural crest. Our findings showed significant differences in the velocity and directionality of GFP+ cells between the NTC and *mgat4b* mut. In the mutant, the cells exhibited significantly lower velocity compared to NTC cells, and their directionality was also impaired (*SI Appendix*, Fig. S5 *A* and *B*). Thus, underscoring the importance of *mgat4b* in facilitating the migration of melanocytes along their paths while maintaining the correct directional orientation.

To explore the fate of these arrested cells, we assessed the survival of these cells by Annexin V staining. A significant and consistent increase (from 18 % to 26%, *P* = 0.0138) in the early apoptotic cell population was observed in *mgat4b* mut animals compared to NTCs (Fig. 3*C*). Furthermore, acridine orange staining was performed to examine the spatial distribution of these apoptotic cells. We observed a markedly higher frequency of fluorescent puncta in the trunk region of *mgat4b* mut animals compared to control (Fig. 3*D* and *SI Appendix*, Fig. S5*C*). This region corresponds to where melanophores were arrested during their migration (Fig. 3*B*). Hence it is likely that the failure to achieve the appropriate spatial positioning and an independent, or a consequent loss of growth factor signaling within this critical time window may preclude the migratory cells from establishing the McSC pool. Since *mgat4b* mutants showed a decrease in both embryonic lateral and adult melanophores, we hypothesized that the McSC pools are altered in these animals. Lateral and adult melanophores arise from an McSC pool that resides in dorsal root ganglion (DRG) (1). We set out to identify McSCs in 4 dpf animals as previously reported (4, 7, 25), and observed multiple dendritic *mitfa*:gfp cells in the *mgat4b* mutant animals, in place of the few elongated McSCs associated with the DRG observed in the NTC animals (Fig. 3 *E* and *F*). These defects extended into one month old animals in which the stripe establishment is incomplete, the mutants display dendritic melanophores and are unable to orchestrate into the stripes (Fig. 3 *G* and *H*). In the 6-mo-old adult animals, while the stripes could be observed, the melanophore numbers were diminished in the *mgat4b* mutant group compared to the NTC group (Fig. 3 *I* and *J*). Therefore, *mgat4b* appears to have a role in establishing the McSC pool. To investigate this further, we resorted to melanophore regeneration assay using [4,(4-Morpholinobutylthio) Phenol] (MoTP) as the melanocyte ablating agent (26). After regeneration, the mutant fish have a lower number of regenerated melanophores compared to the wild type siblings, confirming a defect in the establishment of McSC pool (*SI Appendix*, Fig. S5 *D* and *E*).

### MGAT4B Expression in Melanocytes Is Governed by MITF.

As *mgat4b* plays a key role in melanophore development, we assessed the changes in *mgat4b* expression in melanocytes across the developmental time-window. Reanalysis of zebrafish microarray data from our group showed that similar to the *mitfa* expression, *mgat4b* expression peaks during the 34-36 hpf time-frame (*SI Appendix*, Fig. S2*H*, GSE189059). This coincides with the migration and positioning of melanophores within embryonic stripe patterns in zebrafish development. Given that Mitfa is a key transcription factor responsible for these processes, we hypothesized that *mgat4b* may mediate its effects through a Mitfa program in melanocytes.

To test this, we first assessed whether Mitfa overexpression could rescue defects observed in the *mgat4b* mutant. We used the minicoopR vector, to specifically express *mitfa* in melanophores while also achieving targeted gene manipulation in these cells. To evaluate *mitfa* expression, we injected both this MinicoopR vector and the modified version, where the *mitfa* minigene was replaced with GFP to traced melanocytes into Assam Wildtype (ASWT) embryos. Our analysis revealed a significant increase in *mitfa* transcript levels at approximately 28 hpf in MinicoopR-injected embryos compared to those injected with the modified MinicoopR vector and noninjected controls (*SI Appendix*, Fig. S6*A*). When *mgat4b* was targeted using this construct by the validated sgRNA, we observed defects in melanocyte patterning and reduced melanophore survival, despite the increased *mitfa* expression quantified by qRT-PCR (*SI Appendix*, Fig. S6 *A*–*E*). Suggesting that the effects of Mgat4b are either independent of Mitfa or likely occur downstream of Mitfa in melanocytes. Indeed, studies have shown that glycosyltransferases are regulated by cell-type-specific transcription factors within precise spatiotemporal windows to perform specific functions (27). Thus, we reasoned that *mitfa* could regulate *mgat4b* in melanocytes. To investigate this, we analyzed previously published single-cell RNA sequencing data obtained from zebrafish *mitfa*+ cells at 28 hpf and clustered them based on their levels of endogenous *mitfa* expression (GSM7706846, GSM7706847). Strikingly, we observed a consistent correlation between the expression levels of *mitfa* and *mgat4b* within these cells, suggesting that cells exhibiting high *mitfa* expression also display elevated levels of *mgat4b* (*SI Appendix*, Fig. S6*F*). To test whether *mitfa* transcriptionally regulates *mgat4b*, we overexpressed MITF in B16F10 mouse melanoma cells and assessed its effects on MGAT4B levels. MITF overexpression increased the protein levels of MGAT4B and decreased when MITF was downregulated (*SI Appendix*, Fig. S6 *G* and *H*). Finally, we utilized ChIPqPCR to confirm the binding of MITF to the MGAT4B promoter region. This showed an enrichment of MITF in the MGAT4B promoter region (*SI Appendix*, Fig. S6*I*). In conclusion, our findings demonstrate that MITFA transcriptionally regulates MGAT4B in melanocytes, playing a crucial role in melanophore migration, positioning, and survival.

### Loss of *Mgat4b* Reduces Invasiveness and Disrupts the Velocity and Directionality of Migrating Melanocytes.

**Fig. 4.**
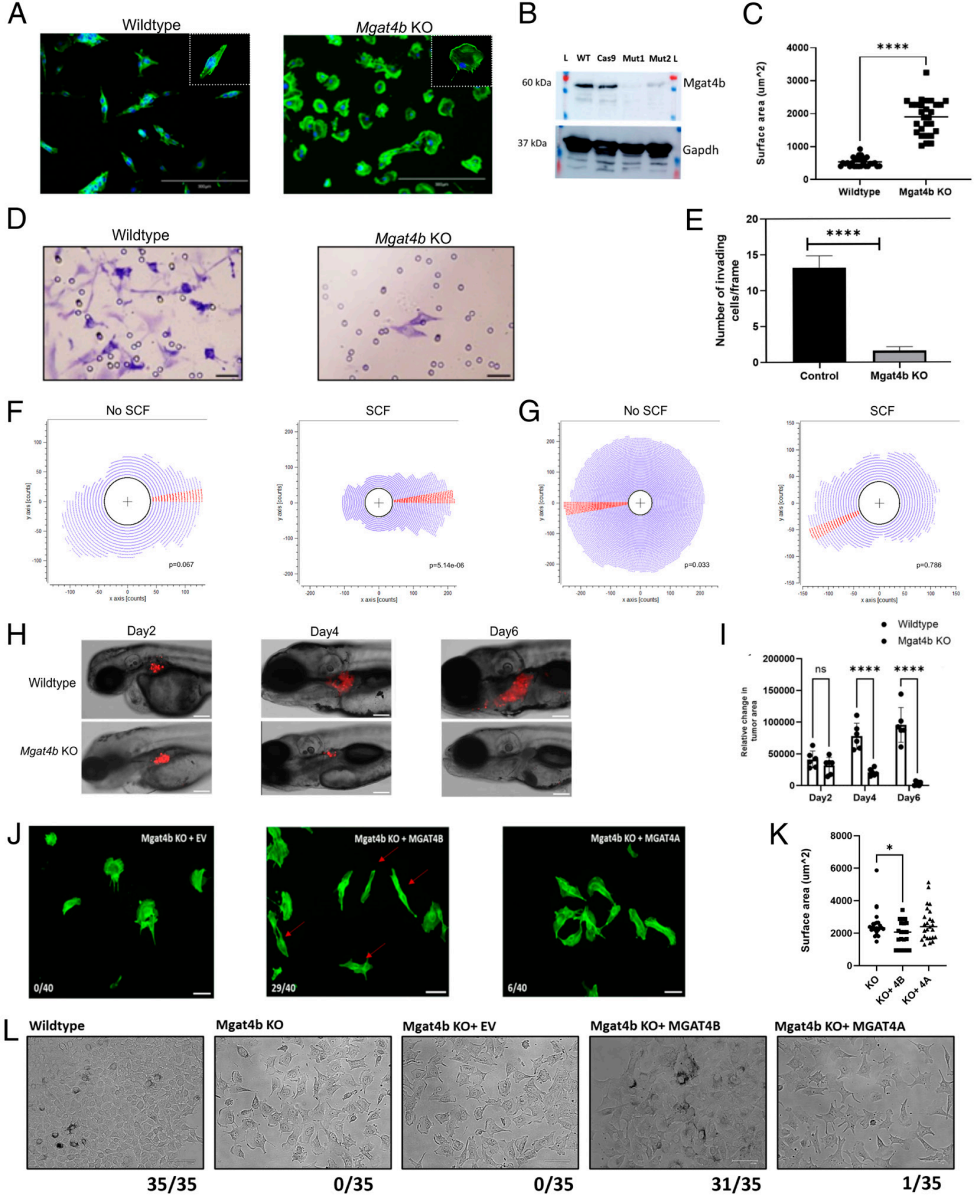
Loss of *Mgat4b* leads to reduced invasion capability and affects directionality of migrating melanocytes. (*A*) Phalloidin staining shows actin filament distribution in wildtype control and *Mgat4*b knockout (Mut1) cells. (Scale bar, 300 µm.) Insets display magnified view of individual cells from each sample. (*B*) Western blot shows protein levels of *Mgat4b* in wildtype control and two clones of *Mgat4b* KO (Mut1 and Mut2) along with Cas9 control in B16-mouse melanoma cells. Mut1 (colony 6) and Mut2 (colony 7) are two independent single-cell clones of Mgat4b mutant B16 mouse melanoma cells. All further validations represented in the manuscript pertain to Mut1 (colony 6), which was used for all subsequent experiments described in the study. (*C*) Scatter plot depicting total cell surface area for wildtype and *Mgat4b* KO cells. (*D*) Toluidine blue label cells on Matrigel depicting the invasion capability of wildtype control and *Mgat4b* knockout(Mut1) cells. (Scale bar, 50 µm.) (*E*) Bar graph depicting invasion of wildtype control and *Mgat4b* knockout cells per frame (Mut1). (*F* and *G*) Rose plot depicting spatial distribution of wildtype control and *Mgat4b* knockout cells(Mut1) in chemotaxis chamber assay, without and with stem cell factor (SCF). (*H*) Xenograft of mouse melanoma cells (wildtype control and *Mgat4b* knockout(Mut1) cells) into the 2dpf zebrafish Perivitelline Space (PVS). Red arrows indicate cells with spindle shaped wild type–like morphology. (*I*) Bar plot shows relative change in tumor area of wildtype control and *Mgat4b* knockout (Mut1) cells. (Scale bar, 50 µm.) (*J*) Phalloidin staining shows actin filament distribution in *Mgat4b* KO(Mut1) cells complemented with Empty vector (EV), *Mgat4b,* and *Mgat4a* constructs. (Scale bar, 50 µm.) Red arrows represent KO cells which regained wildtype like epithelial morphology. (*K*) Scatter plot representing cell surface area of *Mgat4b* KO cells complemented with EV, *Mgat4b* and *Mgat4a* constructs. (*L*) Brightfield images showing cells morphology and colony formation in *Mgat4b* KO(Mut1) cells complemented with EV, *Mgat4b,* and *Mgat4a* constructs along with wildtype and *Mgat4b* KO cells (No transfection control), the numbers listed under each image indicate how many out of 35 colonies exhibit the shown phenotype like wildtype (cohesive-aggregated colony). (Scale bar, 175 µm.) *P ≤ 0.05, **P ≤ 0.01, ***P ≤ 0.001, ****P ≤ 0.0001 and ns *P* > 0.05.

To dissect the molecular mechanisms by which *Mgat4b* regulates melanocyte functions, we knocked out *Mgat4b* in B16 mouse melanoma cells (Fig. 4*B* and *SI Appendix*, Fig. S7 *A*–*C*). The *Mgat4b* KO cells displayed distinct morphological differences compared to the control, including fewer cell–cell contacts and noncohesive aggregate colonies (Fig. 4*A* and *SI Appendix*, Fig. S7*A*). Phalloidin staining of filamentous-actin revealed that the KO cells showed extended filopodia, whereas the control cells were more spindle-shaped and firmly adhered to the substratum (Fig. 4*A*). Western blot analysis of two representative colonies, mut1 and mut2, confirmed a reduction in MGAT4B protein levels (Fig. 4*B*). For all subsequent experiments, we used the mut1 clone as it exhibited minimal protein expression.When measured, the total cell surface area of knockout cells was found to be significantly larger than that of wildtype cells (Fig. 4*C*). Since functional enrichment analysis of zebrafish *mgat4b* mut highlighted ECM binding, migration, and cell adhesion as the most enriched pathways, we evaluated the invasive capability of these *Mgat4b* KO cells using a Matrigel-coated trans-well invasion assay. We observed a significant decrease (*P* ≤ 0.0001) in invading cells, reinforcing the hypothesis that *Mgat4b* could be involved in ECM degradation and extravasation (Fig. 4 *D* and *E*). To further explore this, we assessed the migration capability of KO cells using a chemotaxis assay. Wildtype and KO cells were seeded in a trough connected to a reservoir containing SCF (chemoattractant) on one side and control media on the other, creating a stable SCF gradient across the imaging field. We observed that wildtype cells preferentially migrated toward increasing SCF gradient and maintained persistent migration directionality throughout the imaging period (Fig. 4*F*). In contrast, KO cells showed no preferential direction of migration and additionally moved with significantly lower velocity (Fig. 4*G* and Dataset S1). This suggests that *Mgat4b* is crucial not only for the directional movement of melanocytes but the migration ability in itself. In this context, it is intriguing that the *Mgat4b* KO cells put out extended filopodial projections but are incompetent in cell migration

To evaluate whether *Mgat4b* alters migration of mammalian melanocytes in vivo, we conducted xenograft experiments using wildtype and KO cells in zebrafish embryos. We studied the growth of DiI-labeled wildtype and KO cells by transplanting approximately 200-300 cells into the perivitelline space (PVS) of zebrafish embryos, 2 dpf. Images of the transplants were captured, and the cross-sectional areas were quantified at 4 and 6 dpf (Fig. 4*H*). During the observation period, the cross-sectional areas of the KO xenografts gradually decreased, and by the end of 5 d, the KO cells had completely disappeared (Fig. 4*I*). In contrast, control xenografts continued to grow (Fig. 4 *H* and *I*). Hence, based on chemotaxis assay as well as growth of melanocytes *in vivo, Mgat4b* appears to be essential for directionality and velocity of migration. When the proliferation rates in vitro were measured, the results revealed a highly significant reduction in the proliferation rate. While the mean ± SD for the control was 8.16 × 10^5^ ± 0.28 × 10^5^, it was 1.35 × 10^5^ ± 0.22 × 10^5^ for the *Mgat4b* KO cells (students *t* test *P* < 0.0001, n = 3) compared to the wildtype.

### Profiling of Differential Glycosylation by Lectin Blotting.

It is anticipated that these effects are a result of aberrant expression of glycoproteins, we suspected global alterations in *N*-glycosylation of cell surface proteins. To assess the proteins modified by *Mgat4b*, we employed *Datura Stramonium* Lectin (DSL), that selectively binds the branch generated by MGAT4 activity. We first performed lectin blot analysis on both wildtype and *Mgat4b* knockout B16 mouse melanoma cells, where the available cell quantity allowed for effective enrichment and subsequent target identification. We confirmed reduced DSL binding in the KO cell lysate compared to the wildtype, and this was supported by the FACS based assessment of cell surface labeling (*SI Appendix*, Fig. S7 *D* and *E*).

In the knockout cells we attempted to restore functionality by introducing exogenous MGAT4B, and included MGAT4A for comparison. Q-PCR-based validation showed elevated transcript levels of *Mgat4a* and *Mgat4b* three days after complementation with the respective genes (*SI Appendix*, Fig. S7 *F* and *G*). Surface reactivity to DSL, an indicator of MGAT4B activity, showed that both isoenzymes restored levels comparable to wild-type cells (*SI Appendix*, Fig. S7*E*). However, only MGAT4B and not MGAT4A was able to recover the filopodial phenotype and bring about a reduction in the total surface area of the knockout cells (Fig. 4 *J* and *K*). Additionally, colony forming assays with rescued cells showed that MGAT4B, but not MGAT4A, restored the “loose aggregate” phenotype of *Mgat4b* knockout cells (Fig. 4*L*). Since only MGAT4B rescued both the filopodial and colony-forming phenotypes in knockout cells, this strongly indicates distinct substrate selectivity between these two glycosyltransferases (Fig. 4 *J*–*L*).

### Differential Proteomics Identifies Target Proteins of MGAT4B in Melanocytes.

Having established that *Mgat4b* is essential for melanocyte migration, we next sought to identify the cellular targets of glycosyltransferase responsible for this phenotype. We used a lectin-based approach to enrich glycoproteins specifically modified by MGAT4B in melanocytes. We assessed the levels of β1→4 linked GlcNAc sugars on cell surface of control and *mgat4b* mutant zebrafish melanophores at 2 dpf using *Datura Stramonium* lectin (DSL) labeling. DSL has a strong affinity for β1→4 linked GlcNAc sugars, which are synthesized by MGAT4 activity (24). Flow cytometric analysis of surface accessible DSL reactivity was similar in other (non-GFP positive) cell types between the *mgat4b* mutant and NTC samples. However, in consistence with the earlier observed phenotype a two-fold reduction (*P* = 0.0271) was observed in GFP-expressing melanophores (*mitfa*:gfp+) in *mgat4b* mutant compared to control cells (*SI Appendix*, Fig. S7 *H* and *I*). Melanocytes demonstrate high DSL reactivity, suggesting a requirement for MGAT4 activity in these cells. Interestingly, *mgat4b* mutant showed decreased melanocyte-specific DSL reactivity evidenced by the GFP and DSL double positive cells (*SI Appendix*, Fig. S7 *H* and *I*). Therefore, a DSL-based enrichment strategy would be an effective way to enrich for MGAT4B targets, allowing us to compare wildtype and *Mgat4b* KO cells to reveal the specific targets of this glycosyltransferase in melanocytes.

**Fig. 5.**
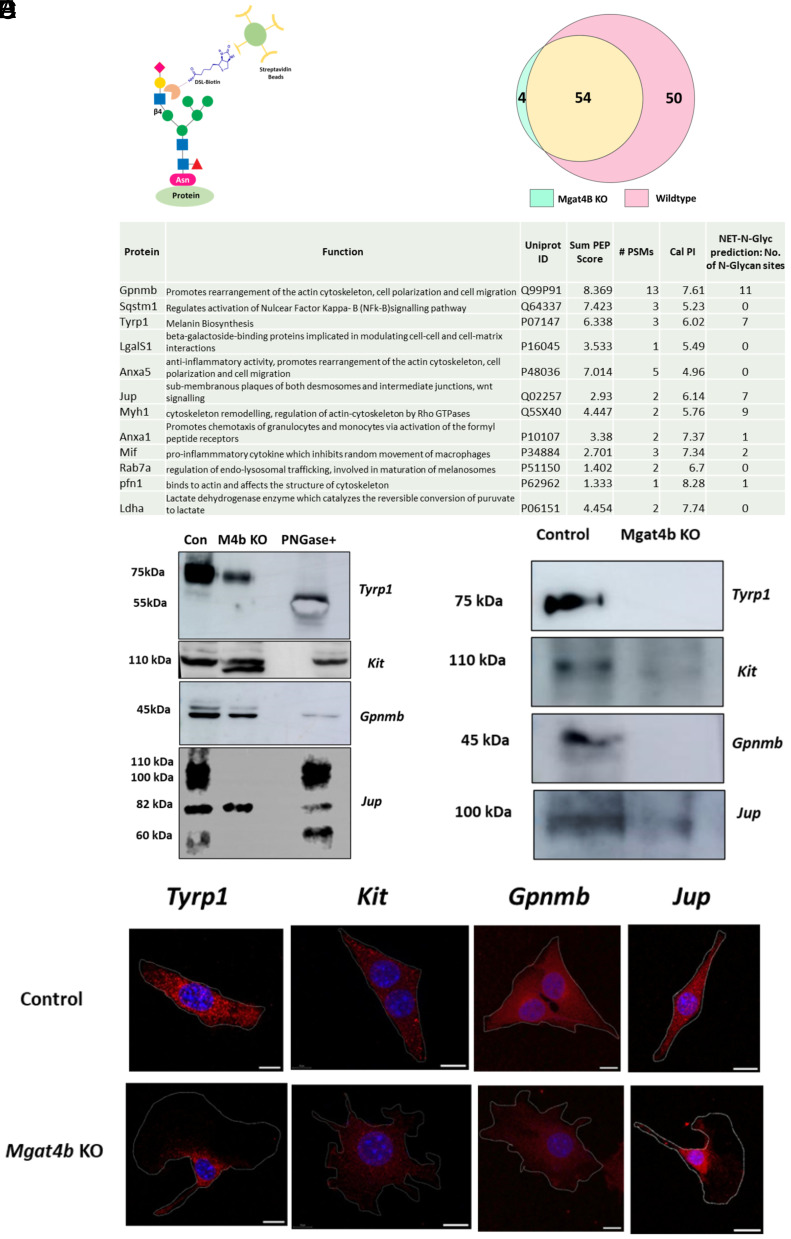
Differential proteomics reveal melanocyte specific target proteins of MGAT4B. (*A*, *B*) Schematic overview of the experimental strategy used to identify downstream targets of MGAT4B in melanocytes, Venn diagram depicting number of proteins identified by mass spectrometry analysis of the DSL-enriched fractions of Wildtype and *Mgat4b* KO proteins. (*C*) List of differentially enriched proteins in wildtype lysates with respect to *Mgat4b* KO along with their Uniprot ID, Sum PEP Score, PSMs, calc. PI and their functional profiling and predicted *N*-glycosylation sites by NetNGlyc—1.0 database. (*D*) Western blot analysis of *Tyrp1, Kit, Gpnmb,* and *Jup* in wildtype and *Mgat4b* knockout (M4b KO) lysate along with PNGase treated wildtype lysate. (*E*) Western blot analysis of *Tyrp1, c-Kit, Gpnmb*, and *Jup* following biotinylated DSL pull-down in wildtype and *Mgat4b* knockout (M4b KO) lysates. (*F*) Confocal microscopy images of immunocytochemistry using *Tyrp1, Kit, Gpnmb,* and *Jup* (red) on permeabilized wildtype and *Mgat4b* KO cells, counterstained by DAPI (blue). The overlay of both channels is depicted. [Scale bar, 50 µm (DSL- *Datura stramonium* Lectin].

While DSL-based enrichment is an effective method for identifying MGAT4 targets in melanocytes, the low abundance of zebrafish cells presents a challenge. Therefore, for identifying MGAT4B targets, we opted to use B16 cells, where the knockout can be combined with DSL enrichment to analyze the glycoproteome affected by MGAT4B (Fig. 5*A*). Differential proteomic analysis was carried out on DSL-enriched proteins from both wildtype and *Mgat4b* knockout B16 cells. Mass spectrometric analysis of DSL-enriched proteins consistently identified 104 proteins in wildtype and 58 proteins in knockout samples with at least one peptide being recognized across two biological replicate experiments. Among these 54 proteins were common between the two samples (Fig. 5*B* and Dataset S3). After applying median filtering and removing common contaminant proteins and associated proteins such as ribosomal proteins, tubulins, and heat-shock proteins, we shortlisted proteins that are putatively modified by MGAT4B (Fig. 5*C*). Based on literature analysis, these proteins may play a role in directional cell migration and invasion. Three of the shortlisted proteins, namely glycoprotein nonmetastatic melanoma protein B (GPNMB), Junctional Plakoglobin (JUP, also called as β-catenin), and tyrosinase-related protein 1 (TYRP1) were further evaluated to confirm whether these proteins are substrates of MGAT4B that could explain the phenotypic outcomes seen in the knockout.

The transmembrane protein GPNMB is a known factor that promotes melanocyte growth and enhances extracellular matrix (ECM) binding (28). Its *N*-glycosylated ectodomain interacts with RGD-containing β1 integrin, leading to focal adhesion formation and subsequent activation of FAK and ERK pathways and controls rearrangement of actin-cytoskeleton, cell polarization, and migration of melanocytes (28, 29). The protein Junction plakoglobin (JUP) is a member of the armadillo protein family, close homolog of β-catenin, and is a component of the desmosome connecting cadherins to cytoskeleton (30). Owing to the lack of signal peptide and its final destination in the cytoplasmic region, JUP does not seem to follow the secretory pathway. A NetNGlyc and NetOGlyc analysis of JUP protein sequence predicted seven and seventeen N-linked and O-linked glycosylation sites respectively, albeit no experimental data are available. TYRP1 is a melanosomal protein involved in the pigmentation process of melanocytes (31). Our earlier data revealed that *Mgat4b* knockout cells were unable to respond to the SCF cue in their environment and failed to migrate toward it. Because of SCF’s critical role in guiding melanocyte movement, its receptor, Kit, a known *N*-glycosylated protein essential for the survival of lateral and adult melanophores in zebrafish (32), was chosen for further validation. Therefore, in addition to the proteins identified in differential proteomics, we examined the tyrosine kinase receptor KIT as it was a compelling candidate for glycosylation modification by MGAT4B.

To determine whether these shortlisted proteins mediate the observed phenotypic effects of MGAT4B, we evaluated their protein levels, relative mobility on SDS-PAGE, ability to bind DSL, as well as the protein localization in wildtype versus *Mgat4b* KO cells. We performed western blots for each candidate with a reference of deglycosylated form using peptide-*N*-glycosidase F (PNGaseF). TYRP1 showed a mobility shift in tune with the known extensive *N*-glycosylation (33). In *Mgat4b* KO cells mobility shift was not observed although the binding to DSL was completely abrogated (Fig. 5 *D* and *E*). However, a reduction in the levels of TYRP1 was observed (Fig. 5*D*). This suggests that although MGAT4B-mediated modification does not significantly affect the overall glycan load, it remains essential for protein stability. Immunolocalization studies further confirmed a decrease in the overall protein levels (Fig. 5*F*). We surmised that the proteins are mislocalised and likely retained within the Golgi apparatus. However, using Phaseolus Vulgaris Leucoagglutinin PHA-L that marks Golgi, IF studies indicated lack of any clear trend in colocalization (*SI Appendix*, Fig. S7*J*). In the *Mgat4b* knockout lysate, the KIT receptor showed two distinct bands around 110 kDa, whereas the wildtype and PNGase-treated lysates displayed only one band of the same size corresponding to the top band observed in the knockout. This suggests a possible proteolytic cleavage of the KIT receptor occurs in *Mgat4b* knockout cells, possibly impairing its ability to respond to the ligand SCF. Immunofluorescence suggested a decrease in the overall KIT levels in the KO and also its localization was nonuniform and restricted to regions around the nucleus. Surface accessibility could not be checked as FACS antibodies recognizing extracellular epitope of KIT did not show immunoreactivity in B16 cells. GPNMB antibody recognized two bands migrating closely around 45 kDa, with the upper band being diminished in the *Mgat4b* knockout lysate and completely disappearing in the PNGase-treated sample. DSL recognized the upper band, indicating GPNMB to be modified by MGAT4B, and this modification is essential for stable expression of this glycoform (Fig. 5*D* &E). Immunofluorescence also indicated an overall decrease in GPNMB expression in the knockout cells (Fig. 5*F*). JUP consistently exhibited three additional bands 110, 100, and 60 kDa along with the main 82 kDa band (Fig. 5*D*). DSL recognizes 100 kDa form which is missing in *Mgat4b* knockout (Fig. 5*E*). The striking absence of 100 kDa band for JUP in *Mgat4b* knockout suggests that JUP has a high probability of nonredundant dependency on MGAT4B for elaboration of the predicated seven *N*-glycan sites. The selective identification of glycoproteins, such as GPNMB, KIT, and JUP which are part of desmosome and adherens junctions, emphasize the role of protein–glycan interactions apart from the well-studied protein–protein interactions in cell–cell interactions.

### MGAT4B Is a Critical Factor in Melanoma Progression.

Having demonstrated the pivotal role of *mgat4b* in early melanocyte development, we set out to assess its potential implications in melanoma tumor initiation. Melanoma cells exhibit a dedifferentiated state similar to stem cells, where they possess heightened plasticity and tumorigenic potential (3). Given Mgat4b’s involvement in orchestrating crucial developmental processes, including cell migration and patterning, there arises a compelling rationale to explore its function within the context of melanoma. Understanding the role of Mgat4b in melanoma may offer valuable insights into the mechanisms underlying tumor progression, metastasis, and perhaps even provide novel avenues for therapeutic intervention aimed at targeting the dysregulated glycosylation pathways characteristic of cancer cells.

**Fig. 6.**
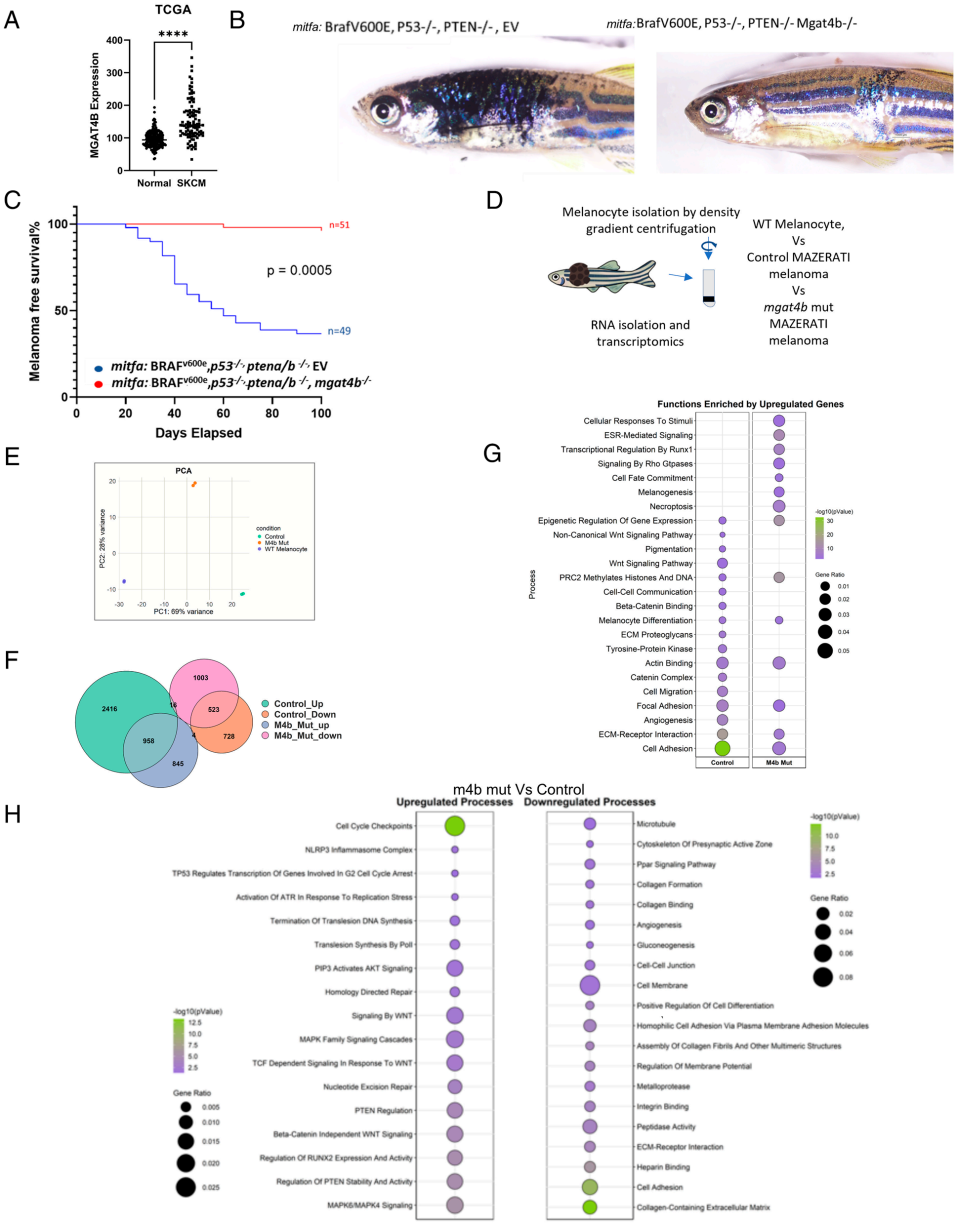
Elevated MGAT4B levels correlate with poor patient survival and are crucial for initiating primary tumors. (*A*) Scatter plot depicting expression of MGAT4B in Skin cutaneous melanoma (SKCM) patients with respect to normal individuals, data procured from TCGA. (*B*) 60 d old Mazerati zebrafish displaying melanoblast-derived tumor developed by combinations of vectors expressing the oncogene BRAF^V600E^, targeting *tp53* and *ptena/b* initiate melanoma *Left*—*mitfa*-BRAF^V600E^, *mitfa*-*p53*^−/−^, *mitfa*-*ptena/b*^−/−^ and *mitfa*:EV, *Right—mitfa*:*mgat4b*^−/−^. (*C*) Melanoma-free survival curves of ASWT zebrafish injected with the indicated combinations of vectors expressing *mitfa*-BRAF^V600E^, *mitfa*-*p53*^−/−^, *mitfa*-*ptena/b*^−/−^ and *mitfa*:EV or *mitfa*:*mgat4b*^−/−^. (*D*) Schematic illustration of the experimental approach adapted to isolate and enrich(percol based gradient) melanophores from one month old wildtype and Mazerati fishes. (*E*) PCA plot depicting relationship between the three samples sequenced. (*F*) Euler plot depicting the number of differentially expressed genes in melanocytes/melanoma cells from Mazerati fishes (control and *mgat4b* mutant (M4b mut) melanoma) with respect to wildtype melanophores. (*G*) Dot plot depicting functional enrichment from the upregulated genes in control and M4b mut melanophores/melanoma cells with respect to wildtype melanophores. (*H*) Dot plot depicting upregulated and downregulated processes in M4b mut melanoma with respect to control melanophores/melanoma *P ≤ 0.05, **P ≤ 0.01, ***P ≤ 0.001, ****P ≤ 0.0001 and ns *P* > 0.05.

To investigate the involvement of Mgat4b in melanoma, we initially examined its expression in skin cutaneous melanoma (SKCM) using the cancer genome atlas (TCGA) database. Remarkably, we observed elevated levels of *MGAT4B* in melanoma patients compared to normal individuals (Fig. 6*A*). Subsequently, by analyzing two distinct datasets that categorized melanoma patients into metastatic and primary stages, we observed a significant increase in *MGAT4B* expression in metastatic patients compared to those at the primary stage (*SI Appendix*, Fig. S8*A*), suggesting that *MGAT4B* may mediate aggressive disease in melanoma. Further exploration into the genetic alterations affecting *MGAT4B* revealed that ~10% of melanoma patients harbor amplifications in *MGAT4B*, which could explain aberrantly high levels of *MGAT4B* expression in affected individuals (*SI Appendix*, Fig. S8*B*). Molecular function enrichment analysis of melanoma, stratified by *MGAT4B* levels, revealed that among other pathways growth factor binding, cytoskeletal protein binding and integrin receptor binding were enriched (*SI Appendix*, Fig. S8*C*). Importantly, patients with high *MGAT4B* expression have poor survival rates, exceeding those of patients with the common BRAF^V600E^ mutation. Our investigation further revealed that melanoma patients with both the BRAF^V600E^ mutation and elevated *MGAT4B* levels have significantly worse survival outcomes compared to those with only the BRAF^V600E^ mutation (logrank test *P* value 0.0152) (*SI Appendix*, Fig. S8*D*). These findings show the association between heightened *MGAT4B* levels and the aggressive nature of melanoma, emphasizing its significance as a prognostic indicator for patient outcomes.

We utilized the well-established Mazerati model to genetically induce melanoma tumors in zebrafish, both with and without targeting *mgat4b* (20). This approach, leveraging the MiniCoopR vector, allowed us to precisely express oncogenes and deactivate candidate tumor suppressor genes within zebrafish melanocytes. As previously established, we injected a cocktail of four plasmids to induce melanoma. This included plasmid expressing the BRAF^V600E^ oncogene and sgRNA targeting the tumor suppressors *p53* and *ptena/b*. We used *mitfa*:Cas9;*mitfa*:gfp plasmid to achieve melanocyte-specific gene ablation and melanoma initiation. Remarkably, the control fishes expressing nontargeting sgRNA rapidly developed aggressive tumors within a month, whereas the *mgat4b* mutant animals failed to develop melanoma altogether. Although melanocytes in the *mgat4b* mutant group showed signs of aggregation, they failed to initiate tumor formation demonstrating the indispensable role of *mgat4b* in initiating melanoma tumors in this zebrafish model (Fig. 6 *B* and *C*).

We explored the mechanisms that prevent mutant cells from initiating melanoma. To do this, we isolated mature melanophores from wild-type zebrafish and melanophores/melanoma cells from the skin of one month old MAZERATI zebrafish with and without *mgat4b* mutations. Cell isolation was done using density gradient centrifugation, followed by RNA extraction and sequencing (Fig. 6*D*). In the wild-type group, we enriched for mature, nontransformed melanocytes, while the NTC and *mgat4b* mutant group included both transformed melanoma cells and melanocytes. Principal component analysis (PCA) of the sequencing data showed clear segregation between the three groups, confirming that the *mgat4b* mutant (M4b mut) cells are distinct from both untransformed wild-type melanocytes and control melanoma cells (Fig. 6*E*). While there was some overlap in gene expression between control and *mgat4b* mutant melanoma cells, most differences were unique (Fig. 6*F*). In control melanoma cells, cell adhesion and migration processes were significantly enriched, but these were not similarly enriched in *mgat4b* mutant melanoma cells (Fig. 6*G*). Consistent with scRNA seq and other experimental data discussed above, compared to control melanoma, *mgat4b* mutants showed upregulation of cell-cycle checkpoint genes and downregulation of cell adhesion and collagen-related extracellular matrix components (Fig. 6*H* and *SI Appendix*, Fig. S9*A* and Dataset S4). We dissected and sectioned the control melanoma and the corresponding melanocyte aggregate from the *mgat4b* mutants. Changes in collagen content using collagen specific staining by Verhoeff-Van Gieson (VVG) stain is not dramatically different, possibly as the mutant is melanocyte specific and bulk of collagen is secreted by fibroblasts in the skin (*SI Appendix*, Fig. S9*D*).

To further validate this observation, we analyzed B16 *Mgat4b* KO cells and conducted a comprehensive proteomic comparison between B16 WT and B16 *Mgat4b* KO cells. Differential proteome analysis revealed a consistent enrichment of pathways related to cell junction assembly, focal adhesion, and cell migration (*SI Appendix*, Fig. S9*B* and Dataset S2). Additionally, the activation of cell-cycle checkpoint genes may contribute to the observed reduction in melanoma growth in the zebrafish mutant. *SI Appendix*, Fig. S9*C* highlights the top differentially regulated proteins between B16 WT and *Mgat4b* KO cells, along with their *N*-glycosylation status and previously identified roles in melanoma. These findings further reinforce the role of MGAT4B in regulating these processes in melanocytes across different model systems. These observations established that ablation of complex glycosylation, such as that mediated by *mgat4b*, could be a viable strategy to deter the initiation of melanoma. To address whether targeting this complex *N*-glycosylation deters the already initiated melanoma we treated the MAZERATI animals with kifunensine, an inhibitor of α-mannosidase I, an enzyme crucial for trimming mannose residues during *N*-glycan processing (34). By blocking this enzyme, kifunensine prevents glycoprotein maturation, causing the accumulation of high-mannose type *N*-glycans (34). Since the MGAT4 enzyme requires a properly trimmed glycan substrate to add *N*-acetylglucosamine (GlcNAc) during the complex glycan processing pathway, kifunensine indirectly reduces MGAT4 activity by limiting the availability of suitable glycan substrates for its function. We first addressed whether kifunensine recapitulates the lateral melanophore phenotype of *mgat4b* mutant. We treated 1 dpf zebrafish embryos with multiple concentrations of kifunensine (50, 100, 150, 200, and 250 µM) for two days. Beyond 200 µM embryo lethality was observed, hence experiments were carried out at 150 µM of this drug (*SI Appendix*, Fig. S10*A*). At this concentration, lateral-melanophore phenotype could be recapitulated (*SI Appendix*, Fig. S10 *B* and *C*). We then resorted to address the possibility of intervention in melanoma. In one month old MAZERATI fishes when the aggregation became visible kifunensine (50, 150 µM) was administered. During the 2 d of treatment, the control vehicle treated animals showed a substantial increase in the tumor area, whereas kifunensine treatment arrested melanoma growth, as indicated by the reduction in tumor area (*SI Appendix*, Fig. S10 *D* and *E*). Suggesting that complex *N*-glycosylation remains an actionable target even after the initiation of melanoma. To selectively inhibit MGAT4B without impacting other MGAT enzymes, we identified FDA-approved drugs with a higher binding affinity for MGAT4B. A virtual screening highlighted several compounds with a significant binding advantage (binding affinity difference > −1 kcal/mol) for MGAT4B compared to related MGATs (*SI Appendix*, Fig. S10 *F* and *G* and Dataset S6). In future, this approach could yield novel therapeutic intervention selectively targeting MGAT4B for use in deterring melanoma growth.

## Discussion

The role of *N*-glycosylation in melanocyte development remains poorly understood. We found that the loss of *mgat4b* in NCC-derived melanocyte precursors impairs their migration, disrupting McSC pool formation and reducing lateral melanophores and melanocyte survival during metamorphosis. This defect affects a specific subpopulation of migratory cells enriched in lectins and adhesion proteins, suggesting that *N*-glycoproteins may guide migration and McSC establishment through interactions with the extracellular matrix. In melanoma, *mgat4b* disruption severely impairs tumor initiation, as transformed cells fail to adhere properly. This is likely due to altered glycosylation of GPNMB, mislocalization of JUP, and dysregulation of adhesion genes, resulting in loose cell aggregates that cannot form tumors. Additionally, *mgat4b* mutation activates cell cycle checkpoints and promotes cell death, mirroring the loss of melanocyte precursors during development. These findings highlight how *mgat4b* regulated developmental processes are reactivated during melanoma initiation.

Though *N*-glycosylation is widespread, *Mgat4b* shows cell-type specific regulation by MITF in melanocytes, mirroring how *Mgat4a* is regulated in pancreatic cells highlighting selective roles for these nonredundant isozymes. This specificity positions MGAT4B as a promising therapeutic target in melanoma. Unlike prior studies that linked FUT8 mediated glycosylation to melanoma metastasis, our work demonstrates a causal role for MGAT4B in tumor initiation. Using kifunensine, we demonstrate that inhibiting complex glycosylation reduces melanoma in zebrafish. However, due to its broad toxicity, a drug selectively targeting MGAT4B could offer a more precise and effective melanoma therapy.

## Materials and Methods

A cell-type specific knockout strategy was used to generate melanophore-specific CRISPR mutants in zebrafish by injecting a *mitfa*:Cas9; *mitfa*:GFP plasmid targeting the *mgat4b* gene. Single-cell RNA sequencing was performed on sorted Mitfa+ve cells to analyze gene expression, and data were processed using Seurat. Melanoma models were created by injecting a cocktail of plasmids overexpressing the Braf^V600E^ oncogene and targeting p53/PTEN tumor suppressors in melanocytes. Glycan profiling of B16 wild-type and KO cells was performed using DSL affinity enrichment, followed by MS/MS analysis to identify Mgat4b-dependent glycoproteins. For additional information, please refer to the *SI Appendix*.

## Supplementary Material

Appendix 01 (PDF)

Dataset S01 (XLSX)

Dataset S02 (XLSX)

Dataset S03 (XLSX)

Dataset S04 (XLSX)

Dataset S05 (XLSX)

Dataset S06 (XLSX)

Movie S1. Time lapse imaging of 28 hpf zebrafish injected with *mitfa*:Cas9; mitfa:GFP: U6 sgRNA non-targetting plasmid as a control group.

Movie S2. Time lapse imaging of 28 hpf zebrafish injected with *mitfa*:Cas9; *mitfa*:GFP: U6 *mgat4b* sgRNA plasmid as test group.

## Data Availability

Transcriptomic data pertaining to single cell sequencing and RNA sequencing has been submitted. Proteomic data pertaining to Lectin Affinity enrichment and whole cell expression from B16 WT and B16 *Mgat4b* KO cells submitted to ProteomeXchange Consortium. Data have been deposited in GEO repository, PRIDE partner repository [GSE278653 (35), GSE278654 (36), PXD056636 (37), PXD056675 (38)].
