# Supplementary material for: Mgat4b-mediated selective N-glycosylation regulates melanocyte development and melanoma progression
Source: Proc Natl Acad Sci U S A. 2025 May 27;122(22):e2423831122. doi: 10.1073/pnas.2423831122 (PMC12146715; doi:10.1073/pnas.2423831122)
Supplement: Supplementary file 1 — Appendix 01 (PDF) [file pnas.2423831122.sapp.pdf]

PNAS

Supporting Information for

Mgat4b mediated selective N-glycosylation regulates melanocyte development and melanoma progression

Babita Sharma[1,2], Keerthic Aswin[1,2], Tanya Jain[3], Ayesha Nasreen[1,2], Ayush Aggarwal[1,2], Yogaspoorthi J Subramaniam[1,2], Jeyashri Rengaraju[1,2], Srashti Jyoti Agrawal[1,2], Mayank Bhatt[1], Bhashkar Paul[1], Koushika Chandrasekaran[1], Aanchal Yadav[1,2], Jyoti Soni[1,2], Rajat Ujjainiya[1,2], Md Quasid Akhter[1], Rajesh Pandey[1,2], Shruthy Suresh[1,2], Srinivasa-Gopalan Sampathkumar[3] and Vivek T Natarajan[1,2]*

*Correspondence should be addressed to
Vivek T Natarajan, PhD.
Email: tnvivek@igib.in, tn.vivek@igib.res.in

Materials and Methods

Zebrafish strains and maintenance

A zebrafish (Danio rerio) breeding colony was maintained at 28.5°C under a 14-hour light/10-hour dark cycle. The wild-type strain Assam WT (ASWT) and Tyrp1 reporter, Tg(f*tyrp1*: GFP) zebrafish lines were used for morpholino injections and were maintained according to standard zebrafish husbandry protocols. f*tyrp1*: GFP plasmid was a kind gift from Dr Xiangyun Wei [1], University of Pittsburgh School of Medicine, and the transgenic line as reported previously [2]was created at CSIR-IGIB zebrafish facility using tol2 transposase microinjections in ASWT line. Phenylthiourea (PTU;0.003%) was added to embryo water before 24 hpf to prevent melanin from masking the GFP fluorescence.

Imaging

Bright-field imaging for live imaging and gene expression pattern (WISH) studies of zebrafish embryos was performed using a Zeiss stereo microscope (Stemi 2000-C). A Zeiss Axioscope A1 Microscope (with AxiocamHRc) was used for fluorescence imaging. Zeiss proprietary software was used to capture images, which were processed and analyzed in ImageJ software. Epinephrine treatment was given to adult fishes as described previously [3] and then imaged.

Morpholino knockdown of zebrafish *mgat4b*

Antisense morpholino was synthesized from Gene Tools against *mgat4b* of zebrafish. The splice-block morpholino used in the study is ACATTTTGATCTTACCTCCGCAATG. Standard control was obtained from Gene-tools and injected at same dosage as *mgat4b* morphlino**.**

Estimation of melanin content

Dorsal images of Control and *mgat4b* morphant embryos were taken at 2 dpf. The images were imported to ImageJ, and mean gray values were taken for melanophores of each embryo set. Mean gray values are inversely proportional to the melanin content of the cell. Corresponding values were then plotted using GraphPad Prism.

mgat4b CRISPR mutant generation in zebrafish

The design of sgRNAs targeting *mgat4b* was conducted utilizing the CRISPRscan online tool. Candidates with a score exceeding 50 in CRISPRscan were selected. Subsequently, these chosen sgRNAs were assessed for potential off-target effects in zebrafish using the CasOFFinder online tool. Two or three of the highest-scoring CRISPRscan candidates, which exhibited no off-targets with ≤ 3 mismatches, were ultimately chosen for further investigation.

Chosen sgRNAs were annealed with the universal reverse primer provided by the CRISPRscan tool and then amplified. PCR purification of the sgRNA amplified product was performed using Qiagen PCR purification kit. IVT (in vitro transcription) of the PCR purified product was performed using Invitrogen T7 Megascript kit. The sgRNA+Cas9 cocktail was microinjected in zebrafish 1 cell stage embryos. The cocktail used for standard injections was: sgRNA – 55pg/nL, Cas9 – 330pg/nL, KCl – 330mM. These injected embryos were tested for the efficiency and presence of mosaic mutations using high-resolution melt analysis (HRMA) assay. For HRMA assay, from each injection batch, DNA was isolated from 8 random embryos and used as a template to set up a PCR with primers flanking the PAM site of each sgRNA. The presence of mutation will lead to formation of heteroduplexes during the PCR which will have low melting temperature compared to the non-injected control (NIC) embryos. Only those batches (putative Fos) were grown for each which had the presence of mosaic embryos in at least 2 out of 8 randomly selected embryos. The putative Fos were grown for each selected sgRNA. After these embryos attained the breeding age, they were set up for breeding with wildtype embryos. For each cross, the offspring embryos were collecting and at 5 days post fertilisation, DNA was isolated from randomly 8 embryos and high-resolution melt analysis (HRMA) was performed to confirm the F0s. The clutch in which there was observable HRMA peak shift or a CT difference of ≥ 0.5 compared to the wild type embryos were set up for growing as putative F1s. After these putative F1 fishes were grown and reached breeding stage (3 months old), they were crossed with wildtype fish and their fins were clipped after breeding. DNA isolated from the fins was used as a template for HRMA to confirm the presence of heterozygosity in the embryos and confirmation of the F1 parent fish genotype. The crosses for which HRMA came out to be positive were grown as the F2 batches to obtain homozygous mutants.

Melanophore-specific CRISPR mutant generation

The cell-type specific knockout strategy was adapted using MinicoopR vector in which *mitfa* promotor (melanocyte specific promotor) drives Cas9 and *mitfa* mini gene and has space for two sgRNA under Ubiquitous (U6) promotor [4]. The *mitfa* mini gene was replaced with GFP using restriction digestion approach. The *mitfa*: Cas9, *mitfa*: gfp plasmid was injected a little ahead of one cell stage in zebrafish embryo and imaged at 2dpf stage. Two sgRNAs were designed using CHOPCHOP (https://chopchop.cbu.uib.no/) targeting *mgat4b* gene. The cloning was performed by digesting the plasmid using BseRI enzyme. We confirmed the clones using PCR based approach using Forward primer specific to plasmid and sgRNA as a reverse primer. The

PCR validated clones were sanger sequenced and then injected at one-cell stage in Zebrafish embryos and the imaging was done at various developmental stages.

Flow cytometry

Cell counts for Mitfa:gfp cells and ASWT embryos (for melanophore counts) were performed using an BD FACSAria II instrument flow cytometer. Briefly, embryos were dechorinated using pronase (5 mg/ml) (Sigma-Aldrich, P8811) for 10-15 min and collected in microfuge tubes. The embryos were deyolked in ice cold Ringer's solution using a micropipette tip and spun at 100 g for 2 min in a tabletop centrifuge at 4°C (Eppendorf, 5418R). The supernatant was discarded and the embryo bodies were trypsinized using TrypLE Express (Thermo Scientific, 12604039) for 15 or 30 min for 24 hpf or 48 hpf, embryos respectively, at room temperature. The cell suspension was passed through a 70 μm cell strainer and washed twice with ice cold phosphate-buffered saline. The cell suspension was analyzed and sorted using the imaging flow cytometry system.

Surface labelling of zebrafish cells: The cell suspension was incubated with the APC or FITC labelled Datura Stramonium lectin (BioPLUS GlycoMatrix DSL- Texas Red (21761014-1), DSL-Fluorescein (FL-1181-2)) for 1 hour on ice and washed three time for 5 minutes each. Cells were analysed using BD FACSAria II instrument flow cytometer and later analysed using FlowJo™ (TreeStar).

B16 mouse melanoma culture

The B16 mouse melanoma cell line was cultured in DMEM-high glucose medium (Gibco, Life Technologies) supplemented with 10% fetal bovine serum (FBS; Gibco, Life Technologies). The cells were maintained in a 37°C incubator with 5% CO_2.

Phalloidin staining

Cells cultured to a confluence of 50,000 cells per 6 well plate for 20 mins at 37°C. Immediately after the incubation the cells were washed with 1x DPBS, twice and then fixed with 4% paraformaldehyde at 37°C. Once fixed the cell were either stored in DPBS at 4°C or taken ahead for F-actin staining with Phalloidin (Alexa Fluor 568 Phalloidin- A22283, ThermoFisher scientific). Phalloidin stained coverslips were then imaged at 20X or 40X with EVOS M7000 Imaging System, Invitrogen.

Colony formation assay

For the colony formation assay, B16 cells (control and Mgat4b ko cells) were seeded at a very low density of 100 cells/cm2 and cultured for appropriate number of days as per the experimental requirement, for a maximum of 7 days. Colonies formed from a single cell were imaged at day7.

Immunofluorescence

The cells plated on coverslips were washed twice with 1× PBS and then fixed with 4% PFA at 37°C for 20 min. The cells were again washed twice with 1× PBS and permeabilized with 0.01% Triton X-100 (Sigma). The cells were blocked with 5% normal goat serum (Jackson Laboratories) overnight at 4°C. The cells were washed twice with PBST (1× PBS with 0.01% Tween-20). The cells were then incubated with 1:100-250 dilution of Tyrp1 (abcam, ab178676), Kit (thermo, 14-1172-82), Gpnmb (abclonal, A1427), Jup (Gamma catenin, PG-11E4) in a moist chamber for 1 h at room temperature.

Incubation with secondary antibody Alexa fluor 594 Molecular probes, Thermoscientific) was performed at room temperature for 1 h. The cells were again washed with PBST. The cells were then mounted on slides using Antifade slow-fade DAPI (Molecular probes, Invitrogen) and visualized using Confocal Microscope (Leica SP8).

Western blotting

Cells were trypsinized with 0.1% trypsin and the pellet was washed twice with 1× phosphate-buffered saline (PBS; Gibco Life Technologies). NP-40 lysis buffer (Invitrogen) was added to the pellet and was incubated on ice for 30 min with pipetting at interval of 10 min. The cells were centrifuged down at 11,200 g for 30 min at 4°C (Eppendorf Centrifuge 5415 R). The supernatant was collected and transferred to a fresh microfuge tube. The protein was estimated using standard BCA protocol (Pierce BCA protein assay kit; Thermoscientific). Equal amount of protein from each sample were resolved in 10 or 12% % SDS gel in 1× Tris-glycine buffer. The gel was blotted onto 0.45 µm PVDF membrane (Millipore) at 150 mA for 1 h. 5% Skim milk was used for blocking for 1 h at room temperature. Incubation with primary antibody was performed for overnight at 4°C. Primary anti-body Tyrp1 (abcam, ab178676), Kit (thermo, 14-1172-82), Gpnmb (abclonal, A1427), Jup (Gamma catenin, PG-11E4), Mgat4b (abclonal, A12810). After washing the blot with 1× TBST, the blot was incubated with HRP-conjugated secondary antibody for 1 h at room temperature. After washing with 1× TBST, the blot was developed using ImageQuant™ LAS 500 chemiluminescence instrument. Densitometry analysis was performed using ImageJ software.

Zebrafish lysates were as previously described [5]and SDS-PAGE was performed as described above.

Reanalysis of 5dpf Sox10+ scRNA sequencing data

Single cell RNA sequencing data of 5dpf *sox10*+ zebrafish cells capturing multiple *sox10* expressing neural crest lineages, including melanophores. Data was analyzed using Seurat v4 and mimics the authors analysis. (**Ref: GSE131136)**

Single-cell sequencing and analysis

Mitfa+ve cells were sorted by FACS from 36 hpf zebrafish. The single-cell RNA libraries were prepared for both samples using the 10× genomics Chromium Next GEM Single Cell 30 Reagent Kit v3.1. The library QC and quantification was done using Agilent bioanalyzer HS DNA kit. The libraries were pooled and sequenced on the NextSeq 2000 platform. Raw bcl files were converted to final count matrix using Cell Ranger v6.1.2 software following the tutorial provided on the 10x genomics website.

FastQ files from control and *mgat4b* knockout zebrafish Mitfa+ve cells were aligned using the 10x Genomics CellRanger v7.2 [6] pipeline to zebrafish genome (Ensembl GRCz11). The Gene-cell matrices (Control: 1923, *mgat4b* knockout: 4120) were uploaded on RStudio (R version 4.3)[7] and standard quality control metrics with the Seurat package (v.5.1) [8]. Only cells with total features >200 and mitochondrial gene counts (%) < 5 were considered as high quality and kept for further analyses. Random sampling was performed in *mgat4b* knockout sample to have the same number of cells as in Control sample for comparison between the two samples. The data from both the sample were integrated using 2000 integration anchors with the help of Find Integration Anchors and Integrate Data functions to merge the datasets and remove any possible batch effects. Then, the

Louvain clustering of the integrated dataset was performed with Seurat (v.5.1) using the FindNeighbors and FindClusters functions (dims = 13, resolution = 0.07) after performing linear dimensionality reduction and checking the dimensionalities of the datasets visualized with elbow plots. Data were projected onto 2 dimensional spaces using Uniform Manifold Approximation and Projection (UMAP) [9] using the same dimensionality values listed above.

Cluster specific genes were identified using the FindAllMarkers and FindMarkers function in Seurat with default parameters (Wilcoxon Rank-Sum test that compares a single cluster against the others). Cluster annotations were given referring previously published datasets. Functional enrichment for markers from specific clusters were performed using GSEA in Clusterprofiler package[10] in RStudio.

Plots were generated either using Seurat or ggplot2 [11].

Pseudotime trajectory construction was performed on the integrated dataset using Monocle3 [12-14].

Cell-Cell communication analysis was performed using Nichenetr package [15]. Briefly, the migratory MIX+ cluster was considered as recipient and receptors from this cluster was identified. All the remaining cells were considered as sender and ligands from these clusters were identified. As Nichenetr package is specific for human/mouse data, we used Diopt[16] tool to find orthologs of the receptors and ligands identified from our clusters. These orthologs were used to construct receptor-ligand interaction information for our dataset.

Melanophore Enrichment from Zebrafish Skin and Melanoma Biopsies and RNA sequencing

Zebrafish skin and melanoma biopsies were collected and enzymatically dissociated using Liberase TM (Sigma Aldrich – 5401020001) at a concentration of 0.25 mg/mL in phosphate-buffered saline (PBS). The tissue was manually triturated to obtain a single-cell suspension, which was then filtered through a 70 µm cell strainer and centrifuged at 500 xg for 15 minutes at 4°C. The resulting pellet was resuspended in 2% Fetal Bovine Serum (FBS) in PBS and layered onto a 50% Percol solution [17]. Centrifugation was repeated at 500 xg for 15 minutes at 4°C. After careful removal of the supernatant, the enriched melanophores at the bottom were washed once with PBS, lysed with Trizol, and processed for RNA extraction.

RNA Seq Library Preparation

RNA sequencing libraries were prepared using the Illumina TruSeq® Stranded Total RNA Library Prep Gold kit (cat. no 20020598) following the manufacturer’s protocol (1000000040499 v00). A total of 250 ng of RNA isolated from the enriched melanophores/melanoma cells from zebrafish skin was used as input. Cytoplasmic and mitochondrial rRNAs were depleted using the Illumina Ribo-Zero rRNA removal beads to reduce the abundance of these highly expressed RNA species.

Following RNA depletion, the RNA was fragmented and reverse transcribed to synthesize the first strand of cDNA. The RNA strand was then digested, and second strand cDNA synthesis was performed to create double-stranded cDNA. The 3’ ends of the double-stranded cDNA were blunted, and A-tailing was performed to facilitate ligation of index

adapters. Subsequently, PCR-based amplification was performed to enrich the cDNA libraries.

Libraries were purified and size-selected using AMPure XP beads (Beckman Coulter, A63881). The quality of the libraries, including size distribution, was assessed using the Agilent HS D1000 Screen Tape (5067-5587) on an Agilent 2200 TapeStation, and library quantification was performed with the Qubit dsDNA High Sensitivity Assay Kit. Final libraries were diluted to 2 nM and pooled equimolarly. Sequencing was performed on the Illumina NextSeq 2000 platform using the XLEAP P4 300-cycle sequencing kit, generating paired-end reads (2 × 151 bp) with a final loading concentration of 650 pM.

Data analysis-Raw fastq files for each sample were first adaptor removed and trimmed for high confident base reads using Trimmomatic tool [18]. The trimmed files were further aligned with zebrafish reference genome GRCz11 using STAR aligner (v2.7.8)[19] . Featurecounts tool was used for calculation of raw counts for each gene in the sample [20]. The differential gene expression analysis was performed using DESeq2 package (v1.40.2) in Rstudio[21]. Functional enrichment analysis was performed using DAVID webtool[22] .

Live imaging of Zebrafish embryos

Live imaging of embryos between 28 hpf-36 hpf were done using Leica SP8 STED confocal microscope. Images were captured in xyzt mode at 10x magnification. The embryo was laid laterally to visualize dorso-lateral cell migration in trunk region. The sample preparation was done exactly as depicted by [23].

Annexin V and acridine orange assay

Zebrafish embryos were dechorionated using pronase (SIGMA; Roche). For deyolking, dechorionated embryos were collected in a 1.5ml microcentrifuge tube. 200µl of ice-cold Ringer's solution was added to the embryos and mixed well by pipetting using a 200µl tip. The tubes were centrifuged at 100 rcf for 1 min, supernatant was removed and further 1ml of ice-cold ringer's solution was added to the embryo body pellet. The suspension was mixed gently by inverting the tubes twice and centrifuged at 400 rcf for 1 min and the supernatant was discarded. A single cell suspension was prepared by adding 10ml TrypLE Express (ThermoFisher Scientific; 12604013) to the deyolked embryos in a fresh petri dish. The solution containing embryo bodies were mixed to decrease aggregation. The petri dishes containing the deyolked embryos were incubated at room temperature for 15 mins (<24 hpf) or 30 mins (24 – 30hpf) and were occasionally flushed with 1 ml pipette to aid disintegration of cells. A 70µm cell strainer was placed above a 50 ml falcon and the single cell suspension was passed through to remove cell clumps and other particles >70um. The cell suspension was flushed a few times with the same solution so as to remove the cells adhering to petri dish. The samples were centrifuged at 1500 rcf for 5 mins at 4°C in swinging bucket rotor mode. The supernatant was discarded and the pellet was resuspended in 1ml ice cold 1X phosphate buffered saline (PBS). The cells were centrifuged again at 1500 rcf for 3-5 mins at 4°C; the supernatant was discarded and the pellet was resuspended again in 1X PBS. Harvested cells were treated with the suggested concentration of recombinant Annexin V Pro APC (EBioscience™, Cat No. BMS306APC-100) for 15 minutes, followed by washing with the binding buffer provided in the kit. Subsequently, the cells were analysed using FACS.

Zebrafish embryos were subjected to staining with the vital dye acridine orange to quantify the number of apoptotic cells per embryo. The assay involved immersing the embryos in a solution containing 10 µg/mL of AO (786-2074, G-bioscoences) in E3 media. After a staining duration of 60 minutes, the embryos underwent three consecutive washes in E3 media. Following staining, the embryos were transferred to Petri dishes for imaging purposes.

Chromatin immunoprecipitation and qRT PCR

B16 Melanoma cells at 80% confluence was fixed with 10% formalin (Sigma Cat No HT501128) and incubated at 37°C for 10 min. 2.5 M Glycine was added to the cells and again incubated at 37°C for 10 min. Cells were washed with ice-cold 1× PBS containing protease inhibitors. Cells were then scraped and centrifuged at 112g for 5 min at 4°C. The cell pellet was lysed in SDS lysis buffer (1%SDS, 10 mM EDTA, 50 mM Tris (pH 8.1)) on ice for 30 min. The cells were then sonicated on bioruptor (DIAGENODE) in ice. The chromatin lysate was then estimated for protein content using BCA kit (Pierce). 10 µg of MITF C5 antibody (Abcam) was taken and incubated with Protein G or Protein A Dyna-beads (Thermo-scientific) overnight at 4°C on a rotator. The next day, the sera was cleared and washed with ice-cold dilution buffer (2 mM EDTA, 150 mM NaCl, 20 mM Tris HCl (pH 8)). 500 µg of chromatin lysate was added to the beads and the final volume was made up to 750 µl using the dilution buffer and incubated for 6 h at 4°C in a rotator.10% of the lysate was kept separately as input. After incubation, the magnetic beads were washed with low salt buffer (0.1% SDS, 1%Triton X-100, 2 mM EDTA, 20 mM Tris HCl (pH 8), 150 mM NaCl), high salt buffer (0.1% SDS, 1% Triton X-100, 2 mM EDTA, 20 mM Tris HCl (pH 8), 500 mM NaCl), and LiCl buffer (0.25 M LiCl, 1%Igepal C-630, 1 mM EDTA, 10 mM Tris HCl (pH 8), 1% deoxy-cholate). Finally, the magnetic beads were incubated overnight at 65°C in elution buffer (1% SDS, 0.75% sodium bicarbonate) and 1 µl of 20 mg/ml proteinase K (Sigma) for elution and subsequent reverse cross-linking. The magnetic beads were separated from supernatant and column purified using Qiagen PCR purification kit, the input control was also included in the purification step. SYBR qRT-PCR was setup using 5 µl of eluted DNA, and graphs were plotted fold enrichment.

CRISPR based mutagenesis of *Mgat4b* in B16 mouse melanoma cells

Guide RNA for each target genes were designed with CRISPRscan online tool. Chosen sgRNAs were annealed with the universal reverse primer provided by the CRISPRscan tool and then amplified. PCR purification of the sgRNA amplified product was performed using Qiagen PCR purification kit. IVT (in vitro transcription) of the PCR purified product was performed using Invitrogen T7 Megascript kit.

The cocktail containing 625ng CRISPR-RNA, Cas9 nuclease- 2500ng and lipofectamine-5ul was incubated at 25*c for 10min. RNP complex was added to the wells according to the labels. After 4-5 hours, the transfection was terminated by washing the cells with DPBS and add fresh complete media (DMEM High glucose with 10%FBS and 1x Anti-anti). The RNP transfected cells were harvested with trypsin-EDTA and resuspended in 1x PBS. Singlet Cells were then sorted into 96-well plates with the help of BD FACSAria™. Sorted cells that formed a visible single colony in each well within a week became ready to be passaged 2 to 3 weeks after sorting. These colonies were individually validated for the mutation using Sanger sequencing followed by western blotting.

Regeneration of ablated melanocyte population in zebrafish embryos

NTC and *mgat4b* mutants were allowed to breed and embryos were collected and incubated at 28° C for 24 hours. GFP+ embryos at 1 dpf (1 day post fertilization) embryos were transferred to a 24-well plate (5 embryos per well). Each well consisted of 100uM (4,[4-Morpholinobutylthio) Phenol] MoTP prepared in E3 water from a stock solution of 10mM with a total volume of 2 mL in each well. MoTP is changed on the subsequent day (2dpf) and washed out after 3 days of treatment (4dpf). Fishes are kept in E3 water with a change of water after every 24 hours and the regeneration of melanocytes was tracked across samples until 8dpf stage. Fishes were anesthetized using 1X tricaine (from a stock of 20X) and placed over 5% methyl cellulose for imaging each day after ablation under light microscope. The number lateral line melanophores were noted using brightfield imaging.

Modelling Approach in Zebrafish for Rapid Tumor Initiation (MAZERATI)

Primary melanoma was initiated in ASWT fishes using method discussed in [4]. Plasmids from Addgene 118846, 118850, 118841, 118845 were used for the study. These plasmids will overexpress BrafV600e oncogene specifically in melanocytes and target *p53* and *pten* tumor suppressors again specifically in melanocytes. Addition to this we used modified *mitfa*:cas9;*mitfa*:gfp plasmid to target *mgat4b* in the melanocytes. Cocktail of five plasmids were injected in suggested concentration, in one-cell stage to transform the melanocytes. Aggressive tumors were initiated in the zebrafish within a month.

T7 endonuclease I mismatch cleavage assay

PCR amplicons spanning the *mgat4b* target site were amplified from crude DNA isolated from GFP+ cells sorted out from 2 dpf NTC and *mgat4b* mutant animals using Phusion polymerase (New England Biolabs) and primers sgRNA1 (Fwd-CTGATCGTGGAGTTCATGCT, Rev- CCCCTAATTCTTACCGCATC) and sgRNA4 (Fwd-TGAGGTCACCACCAGTCTAA, Rev- ATGCGTATGAAGTCTCCAGC). PCR products were column purified before 150-200ng of DNA was hetero-duplexed by heating to 98°C for 10 minutes and then slowly cooling to room temperature before placing on ice. Digestions were performed in a volume of 15 µl with 5U of T7 endonuclease I (New England Biolabs) at 37°C for 30 minutes before cleavage was analysed on a 2% agarose gel.

Chemotaxis Assay

Chemotaxis chamber assay was set-up in according to the protocol described by the manufacturer. Briefly, each chemotaxis coverslip has 3 (1mm wide) trough regions where the cells are seeded. Each trough is surrounded by media reservoirs on either side. The trough is connected to media reservoirs on either side by a small opening that allows for gradual diffusion of chemotactic agent across the trough, generating a concentration gradient. Once the cells are adhered and the chemotactic agent is added into the respective reservoir, the chamber is set-up on the microscope stage for imaging. Growth promoting chemokine stem cell factor (SCF, PeproTech #300-07). Time-lapse XYT imaging was done for ~20 hours with time interval of 30mins. During the imaging the slide was maintained at 37°C with 5% CO_2 using Environment controlling unit. Imaging was done with Confocal (SP8) microscope at 10x magnification. Chemotaxis analysis was done by individually generating the tracks of each cell (>50 cell/per experiment) using

manual tracking plugin in Fiji and then processing the track information in chemotaxis and migration tool (Fiji Plugin) developed by ibidi.

Cell Invasion Assay

Cell invasion assay was performed using a matrigel assay system (Corning Biocoat) in a 6-well format. Briefly, 100,000 cells each for wildtype and mgat4b knockout were seeded onto Matrigel-coated invasion chambers as per the manual. The next day, cells were fixed and labelled by Toluidine Blue to stain the invaded cells. The invaded cells were imaged using a brightfield microscope with at least 4 to 6 fields per well, and ImageJ was used to quantify the number of invaded cells. Relative invasion was calculated by normalizing all data to the number of invaded cells in the control group.

B16 mouse melanoma cells Xenotransplantation

B16 mouse melanoma cells were cultured under standard conditions and harvested at 70–80% confluence. Cells were resuspended in sterile PBS at a concentration of 1 × 10^7 cells/mL and labeled with a fluorescent dye (CellTracker™ CM-DiI Dye) for visualization. Zebrafish embryos of the Assam wildtype (ASWT) line were dechorionated at 48 hours post-fertilization (hpf) using fine forceps. Approximately 100–200 cells were microinjected into the perivitelline space of each embryo using a glass capillary needle under a stereomicroscope. Injected embryos were maintained at 34 °C in E3 medium supplemented with 0.003% PTU to inhibit pigment formation. Embryos were monitored daily for tumor cell proliferation and angiogenic response using fluorescence microscopy.

B16 mouse melanoma cells processing followed by LC-MS/MS

Cells were lysed using RIPA buffer and centrifuged at 15,000 g for 15 minutes at 4°C. The supernatant was transferred to a new microcentrifuge tube for proteomics analysis. Protein precipitation involved overnight incubation of the supernatant with four times volume of pre-chilled acetone, followed by centrifugation at 15,000 g for 15 minutes at 4°C. The resulting protein pellets were resuspended in 50 mM ammonium bicarbonate buffer (pH 7.8). Protein quantification was performed using the Bradford assay, and 20 µg of protein from each sample was used for quantitative proteomics analysis (SWATH-MS). In brief, protein samples were treated with PNGase F (V483A) and incubated at 37°C for 4 hours. Following this, samples were reduced by adding dithiothreitol (DTT) to a final concentration of 2 mM and heating at 56°C for 30 minutes. The samples were then cooled to room temperature and alkylated with iodoacetamide (IAA) at a final concentration of 2.2 mM, incubating in the dark for 15 minutes. The samples were digested with trypsin (V5111, Promega) at a 1:20 enzyme-to-substrate ratio for 18 hours at 37°C. Tryptic peptides were purified using Oasis HLB 1 cc Vac cartridges (Waters) according to the manufacturer's protocol, vacuum dried, and stored at -20°C until LC-MS/MS analysis. **Data acquisition:** The tryptic digests from the samples were suspended in 0.1% formic acid and analyzed on a quadrupole-TOF hybrid mass spectrometer (TripleTOF 6600, SCIEX) coupled to an Eksigent NanoLC-425 system using the Sequential Window Acquisition of All Theoretical Mass Spectra (SWATH-MS) method by operating the mass spectrometer in data-independent acquisition mode. Optimized source parameters were used, curtain gas and nebulizer gas were maintained at 25 psi and 20 psi respectively, the ion spray voltage was set to 5.5 kV, and the temperature was set to 300°C. About 4 µg of peptides were loaded on a trap column (ChromXP C18CL 5µm 120 Å, Eksigent, SCIEX) and online desalting was performed with a flow rate of 10 µl per minute for10 min. Peptides were separated on a reverse-phase C18 analytical column (HSS T3, 100 Å, 1.8 µm, Waters) in 55 minute

buffer gradient at a flow rate of 6 μl/minute using water with 0.1% formic acid (buffer A) and acetonitrile with 0.1% formic acid (buffer B) as follows:

Time (min)	%A	%B
0	97	3
38	75	25
43	68	32
45	20	80
45.5	10	90
48	10	90
49	97	3
57	97	3

A SWATH-MS method was created with 96 precursor isolation windows, defined based on precursor m/z frequencies in DDA run using the SWATH Variable Window Calculator (SCIEX), with a minimum window of 5 m/z. Data were acquired using Analyst TF 1.7.1 Software (SCIEX). Accumulation time was set to 250 msec for the MS scan (400-1,250 m/z) and 25 msec for the MS/MS scans (100-1,500 m/z). Rolling collision energies were applied for each window based on the m/z range of each SWATH and a charge 2+ ion, with a collision energy spread of 5. Total cycle time was 2.79 s. **Data Analysis:** Raw file with .wiff extension from SWATH-MS runs were analysed using spectronaut software version 19 (Biognosys) using directDIA workflow. For protein identification, Mus Musculus protein database from uniprotKB with 54,707 entries was used with upto 2 missed cleavages. False discovery rate (FDR) was controlled at 1 % for proteins. Rest of the parameters were kept at Spectronaut Pulsar's default settings, cross-run data normalization was performed where the normalization strategy was set to automatic. Quantitative data was exported in the form of 'Run Pivot Report' and differential protein analysis was performed in Microsoft Excel.

DSL Blotting for glycan profiling

B16 cells (wildtype (WT) and *Mgat4b* KO) were washed with cold DPBS and trypsinized with 0.1% trypsin (Gibco, 15090046 diluted in Versene, Gibco, 15040066). and 10 million cells were counted for each sample. Cell pellets were resuspended in the RIPA buffer according to their size (G-Biosciences-786-490). Protein concentrations of total lysates of WT and *Mgat4b* KO cells were estimated by the Bradford assay [24]. Proteins were resolved through a 7.5 % SDS-PAGE and transferred to nitrocellulose membrane at a constant current of 250 mA for three hours at 4 °C. Membranes were blocked at room temperature with 2.0 % gelatin in 0.1 % PBST (phosphate buffered saline with 0.1 % v/v Tween-20) (10 μL, 1.0 h) followed by incubation with fluorescein isothiocyanate-conjugated Datura stramonium lectin (DSL) in PBST (1:5000 dilution; stock conc. 2.0 mg/mL; 5.0 mL, 1.0 h). Membranes were washed with 0.1 % PBST (10 μL × 6, five min incubation for each wash). The blots were visualized using a digital fluorescence imager (Amersham Typhoon) using a Cy2-filter for excitation. Two biological replicates, each with two technical replicates, were performed for the lectin blotting experiments.

Proteomics study of DSL affinity enriched glycoproteins

Both B16 wild-type and KO cells were cultured in T-75 tissue-culture treated flasks under sterile conditions in DMEM supplemented with 10 % fetal bovine serum (FBS) and 1.0 % pen-strep (50 U/mL of penicillin and 0.05 mg/mL of streptomycin) in a humidified incubator

with a 5.0 % carbon dioxide atmosphere at 37 °C. Once the cells attained 70-90 % confluency, the media containing the floating cells was aspirated. The adherent cells were gently washed with PBS (5.0 mL) once and aspirated. Cells were detached by incubating with 0.25% trypsin (1.0 mL) at 37 °C for 2-5 min and diluted with complete media (9.0 mL). Cells were collected in 15 mL tubes and centrifuged at 500g for five min, supernatants were aspirated, and discarded. Cells were re-suspended in PBS (1.0 mL), cell density was measured using a Z2 particle counter, cells were aliquoted in 1.5 mL microcentrifuges, centrifuged, and supernatants were aspirated. Both wide-type and KO B16-cells (5.0 × 10^6 cells) were suspended on lysis buffer (10 mM tris base, 140 mM NaCl, 1.0 % v/v triton-X-100, and 1.0 mM phenylmethylsulfonyl fluoride in MS-grade water, pH 7.4) (100 µL), probe sonicated (amplitude 25 %; Sonics, Vibra-cell) for 15 min on an ice-bath (amplitude 25 %), centrifuged (16000×g, 10 min, 4 °C), and the supernatant were transferred to fresh pre-labelled tubes. Protein concentrations were estimated using the Bradford assay. Total cell lysates (1.0 mg in 500 mL PBS) were allowed to bind to biotin-conjugated DSL (b-DSL) (5.0 µL from a stock solution containing 2.0 mg/mL) overnight at 4 °C with gentle agitation on an end-to-end rotor. The mixtures were treated with streptavidin-conjugated dynabeads (50 µL of slurry; binding capacity of 10 mg biotinylated antibody/mg beads; pre-washed with PBS (500 µL, thrice)) and incubated at 4 °C for two hours with gentle mixing. Samples were placed on the magnetic separator for 1.0 min, the supernatants were aspirated and discarded. The beads were washed in NP-40 buffer (0.1 % v/v NP-40 in PBS; 100 µL; three times) and the supernatants were aspirated and discarded. The beads were re-suspended in 200 mM *N*-acetyl-D-glucosamine (GlcNAc) solution in PBS (50 µL), vortex mixed for 10 min at room temperature, placed on the magnetic separator for 1.0 min, and the supernatant was carefully aspirated. Beads were subject to a second round of elution with GlcNAc, the supernatants were combined, and processed for sample preparation for mass spectrometry as given below.

Buffers for sample preparation for mass spectrometry

50 mM Ammonium bicarbonate (AMBIC) buffer: Dissolve 3.95 mg of ammonium bicarbonate powder in 1.0 mL of water (mass spectrometry grade) in a clean glass vial; vortex mix thoroughly before use.

200 mM Dithiothreitol (DTT) buffer: Dissolve 30.85 mg of DTT in 1.0 mL of AMBIC buffer in a clean glass vial; vortex mix thoroughly before use.

20 mM Iodoacetamide (IAA): Dissolve 3.7 mg of IAA in 1.0 mL of AMBIC buffer in a clean glass vial; vortex mix thoroughly before use.

Sample preparation for mass spectrometry and bottom-up protein identification

Proteins (100 µl each from WT and KO samples, in 1.5 mL microcentrifuge tubes (Eppendorf)), eluted by GlcNAc from the lectin-affinity enrichment, were treated with ice-cold acetone (900 µL) and allowed to precipitated at −20 °C for 24 h. Samples were centrifuged (16000×g, 4 °C, 10 min) and the supernatants were aspirated and discarded. Protein precipitates were re-suspended in AMBIC buffer (100 µL), heated at 65 °C for 10 min (at a shaking speed setting of 5 on a Torrey-Pines shaker). Samples were treated with DTT (2.5 µL; 200 mM) and heated at 60 °C for 45 min, followed by treatment with IAA (10 µL; 20 mM) at 37 °C for 45 min, on a shaker. Another aliquot of DTT (2.5 µL; 200 mM) was added to the samples and incubated for at 37 °C for 30 min on a shaker [25]. Samples were treated with mass spectrometry grade trypsin (4.0 µL, 2.0 mg, Sequencing grade modified trypsin, Porcine, Promega, Cat. No. V511A) and incubated overnight at 37 °C.

Samples were then treated with formic acid (1.0 µL) and concentrated to dryness in a refrigerated vacuum centrifuge at 4 °C. The samples were re-suspended in 5.0 % v/v aqueous acetonitrile containing 0.5 % v/v formic acid (30 µL) and de-salting was performed using reverse phase C-18 zip tips as given below.

Desalting of tryptic digests using C-18 zip tips:

1. First, acetonitrile (ACN) (10 µL) was used to wet the C-18 zip tips by aspiration using a P10 micropipette
2. Tips were washed using mass spec grade water with 0.1 % formic acid (FA) (10 µL) through aspiration by pipetting.
3. Samples (10 µL) were loaded on the zip tips through aspiration
4. Sample-loaded zip tips were washed using mass spec grade water with 0.1 % formic acid (FA) (10 µL)
5. Samples were eluted using 50 % aq. ACN with 0.1 % FA (10 µL)
6. Samples were eluted for a second time using ACN (10 µL) and pooled in the same tube as in step 5.
7. Samples were evaporated to dryness in a refrigerated vacuum centrifuge at 4 °C.
8. Samples were re-suspended in 5% v/v aq. ACN with 0.1 % FA (12 µL).
9. Samples were subjected to nano-liquid chromatography tandem mass spectrometry (nano-LC-MS/MS) analysis at the NII Central Mass Spectrometry Facility (CMSF).

Data acquisition using nano-LC-MS/MS, data processing, and data analysis

Desalted tryptic digests (5.0 µL per run) were loaded onto Vanquish Neo nano-LC (fitted with PepMap Neo C18 (5.0 mm, 300 mm × 5.0 mm, 1500 bar, trap/guard column and DNV PepMap Neo C18 (2.0 mm, 100 Å, 75 mm × 150 mm) capillary column) connected to an Orbitrap Exploris 240 High-resolution Mass Spectrometer (Thermo Fisher Scientific), equipped with a Nanospray Flex ESI source connected through an Nanobore stainless stell emitter (40 mm length; outer diameter 1/32 inch). Mobile phase A was kept as 0.1 % v/v formic acid in water and mobile phase B was kept as 0.1 % v/v formic acid in 80 % aqueous acetonitrile. Elution was performed using a multi-step gradient of 1.0 % to 99 % of mobile phase B from 0-100 min at a flow rate of 300 nL/min. Mass spectrometry data collection was performed in positive ion mode with electrospray at 1.9 kV, RF lens value at 70%, and ion-spray (1.9 kV) kept at 275 °C. MS1 scans were performed at a range of 375-1200 m/z with orbitrap resolution set to 60,000 and MS/MS scans at the resolution of 14000 in a data-dependent acquisition (DDA) mode. Higher-energy collision dissociation (HCD) was applied at a normalised collision energy (NCE) of 30%. Peptide with charge states of +2 to +6 were selected for fragmentation in MS/MS mode.

Raw data from nano-LC-MS/MS was processed using the XCalibur proteome discoverer (PD 3.0) software with SEQUEST HT as search engine. The acquired data was searched against UniProtKB complete mouse proteome database. Oxidation of Met was set as dynamic modifications and carbamidomethylation of Cys was set as a fixed modification. Parameters were set to a precursor mass tolerance of 10 ppm, fragment mass tolerance of 0.02 Da, and two missed cleavages allowed for trypsin. Proteins and peptide lists were compiled and represented as Venn diagrams using the protein distribution option of PD 3.0; Complete list for protein ID was generated separately for intra-samples (run to run). A list of probable target candidates of *Mgat4b* were identified based on abundance ratio

between control and KO samples. The proteins listed in the table were only present in wildtype samples and were absent in *Mgat4b* KO samples.

For label-free quantification, the processing was done by combining the technical runs for each sample. Label-free quantification (LFQ) was applied to identify relative quantitation of protein in control and KO samples. Label free quantitation was performed based on intensity of the precursor ion. For data processing, protein database was imported in the FASTA file format. The study factors were defined as knockout and wild type in the new study following which the raw data files were added to the study in .raw file format. The technical replicates of wildtype samples were defined as control and the knockout as sample. Under sequest HT node, enzyme selected was trypsin (full) with maximum missed cleavage sites of two, minimum peptide length of six amino acids (aa), maximum peptide length 144 aa, precursor mass tolerance 10 ppm, and fragment mass tolerance of 0.6 Da. Dynamic modification for oxidation (+15.995 Da) at methionine residue and static modification for carbamidomethyl (+57.021 Da) at Cystine residue were defined. Acetylation (+42.011 Da), methionine loss (-131.040 Da) and methionine loss with acetylation (-83.030 Da) at N-terminus were also selected under dynamic modifications (protein terminus). Dynamic modifications are the modifications which may or may not be present while the static modification is applied universally to every instance of the specified residues or terminus.

Plasmid transfections in B16 mouse melanoma cells

B16 cells were trypsinized and seeded at density of 1×10^5 cells/well in 6 well plate (nunc) and incubated overnight in antibiotic containing DMEM + 10% FBS. At the time of transfection, the cells were replaced with serum and antibiotic free media OptiMEM (Gibco, Life Technologies). Lipofectamine 2000 was used at a ratio of 1:3 with pcDNA 3.1(empty vector control) plasmid, Mgat4a and Mgat4b coding sequence containing plasmids. The cells were incubated for 6 hours with the transfection mixture containing Lipofectamine 2000, OptiMEM, and the plasmids. The media was then replaced with antibiotic containing DMEM + 10% FBS and incubated for 72 h, and downstream experiments were performed.

Drug Treatments

Kifunensine (Cas no. 109944-15-2) was dissolved in DMSO and a final concentration of 150 µM was prepared in Embryo water. One-month old Mazerati zebrafish were treated for 2 days with a change of drug on each day.

Imaging and Quantitative Analysis

Images of tumor area was captured using a Nikon SMZ800N camera. Brightfield images were adjusted for contrast and color balance for clarity. To quantify the effect of drug, tumor area was outlined and compared before and after treatment using ImageJ. Percent change in tumor area was calculated as a ratio of the change in tumor area across 2 days to the area covered by tumor after 2nd day of kifunensine treatment. Student's t tests were performed using GraphPad Prism 9.

Molecular Docking

For MGAT4A, MGAT4B, MGAT4C and MGAT5 structure, Alpha-fold model [26]was used since there were incomplete crystal strutures. The Model was pre-processed, polar hydrogen bonds added, kollman charges added and pdbqt structure was generated

using Autodocktools. Grid box was defined for the models using Autodocktools. The ligand library of 2800 FDA approved drugs were energy minimized and converted to pdbqtformat using PyRx tool. Autodock Vina was used to do the virtual screening [27].

Statistical analysis and graphs

Student's t-test was performed to obtain statistical significance in the data. Asterisk on the error bar corresponds to *P ≤ 0.05, **P ≤ 0.01, ***P ≤ 0.001, ****P ≤ 0.0001 and ns P > 0.05. Graphs were plotted using GraphPad prism.

Supplementary Figures

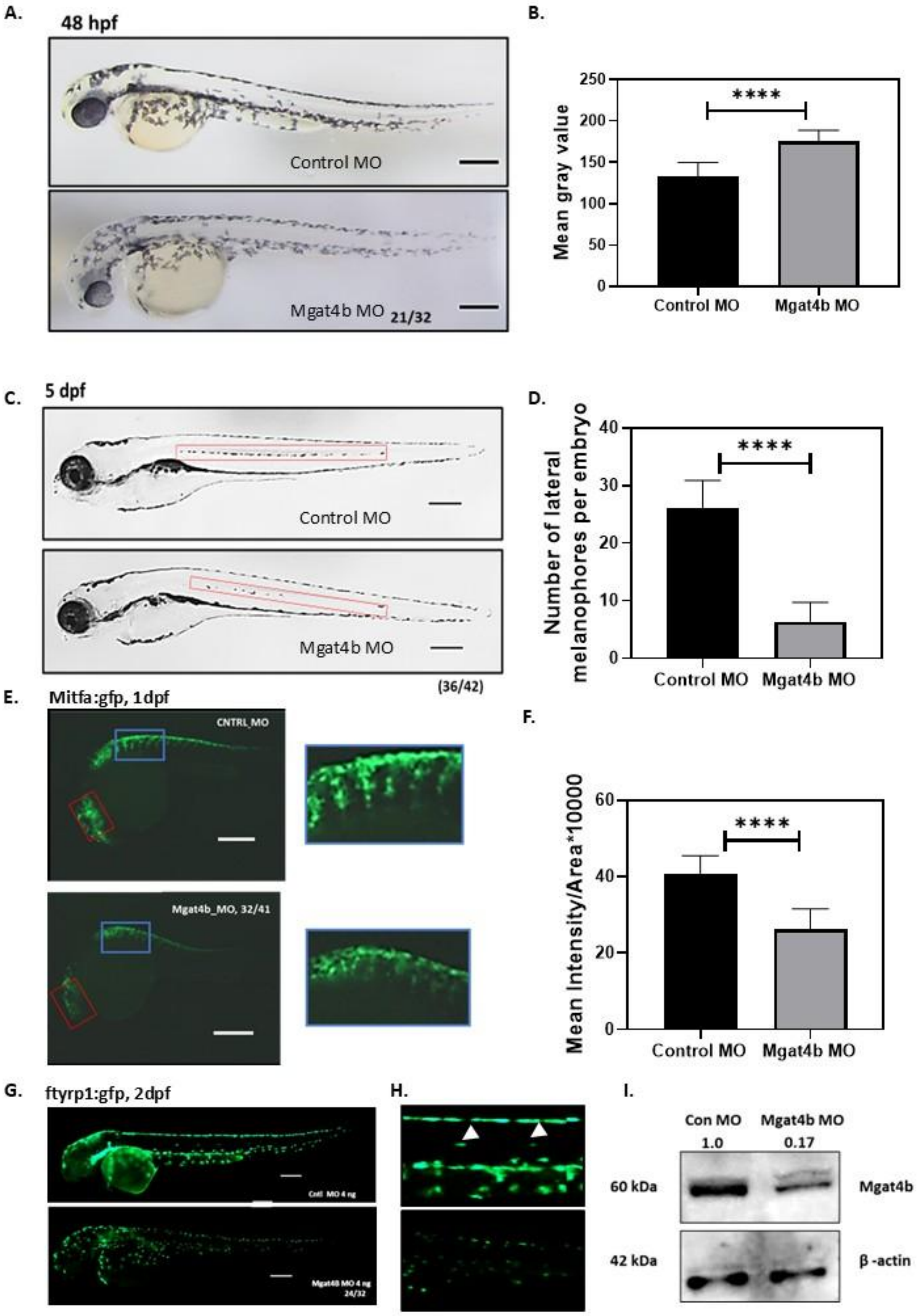
A.
48 hpf
Control MO
Mgat4b MO 21/32
B.
Mean gray value
Control MO
Mgat4b MO
C.
5 dpf
Control MO
Mgat4b MO
(36/42)
D.
Number of lateral melanophores per embryo
Control MO
Mgat4b MO
E.
Mitfa:gfp, 1dpf
CNTRL_MO
Mgat4b_MO, 32/41
F.
Mean Intensity/Area*10000
Control MO
Mgat4b MO
G.
ftyrp1:gfp, 2dpf
H.
I.
Con MO
Mgat4b MO
1.0
0.17
60 kDa
42 kDa
Mgat4b
β -actin

Supplementary Figure 1: Targeting *mgat4b* during early zebrafish development leads to pigmentation and melanophore patterning defects: A. Brightfield images of the lateral view of control and *mgat4b* morphant embryos at 2 days post fertilization (dpf). The black structures observed are melanophores, Scale bar- 100μm **B**. Melanin quantitation from the brightfield images of control and *mgat4b* morphants was carried out using ImageJ platform. The mean gray values are inversely linked to melanin content of the embryo and are represented as a Bar graph **C**. Brightfield images of the lateral view of control and *mgat4b* morphant embryos at 5dpf, Scale bar- 100 μm **D**. The number of lateral mid-line melanophores were counted manually (highlighted in red box) represented in the graph. Scale bar- 100μm **E.** Fluorescent images of control and *mgat4b* morphants at 1 dpf. Red boxes mark the constant region chosen to quantify mean fluorescent GFP intensity per embryo, Scale bar- 100 μm **F.** Mean intensity per area is quantified and depicted in the bar graph **G.** Fluorescent images of control and *mgat4b* morphants at 2 dpf, Scale bar- 100 μm **H.** Zoomed inset of 2dpf *ftyrp*:gfp control and *mgat4b* morphant trunk region. Scale bar- 100μm **I.** Western blot analysis shows Mgat4b protein levels in bulk (100 embryos) of control and *mgat4b* morphants at 2dpf

*p ≤ 0.05, **p ≤ 0.01, ***p ≤ 0.001, ****p ≤ 0.0001 and ns p > 0.05

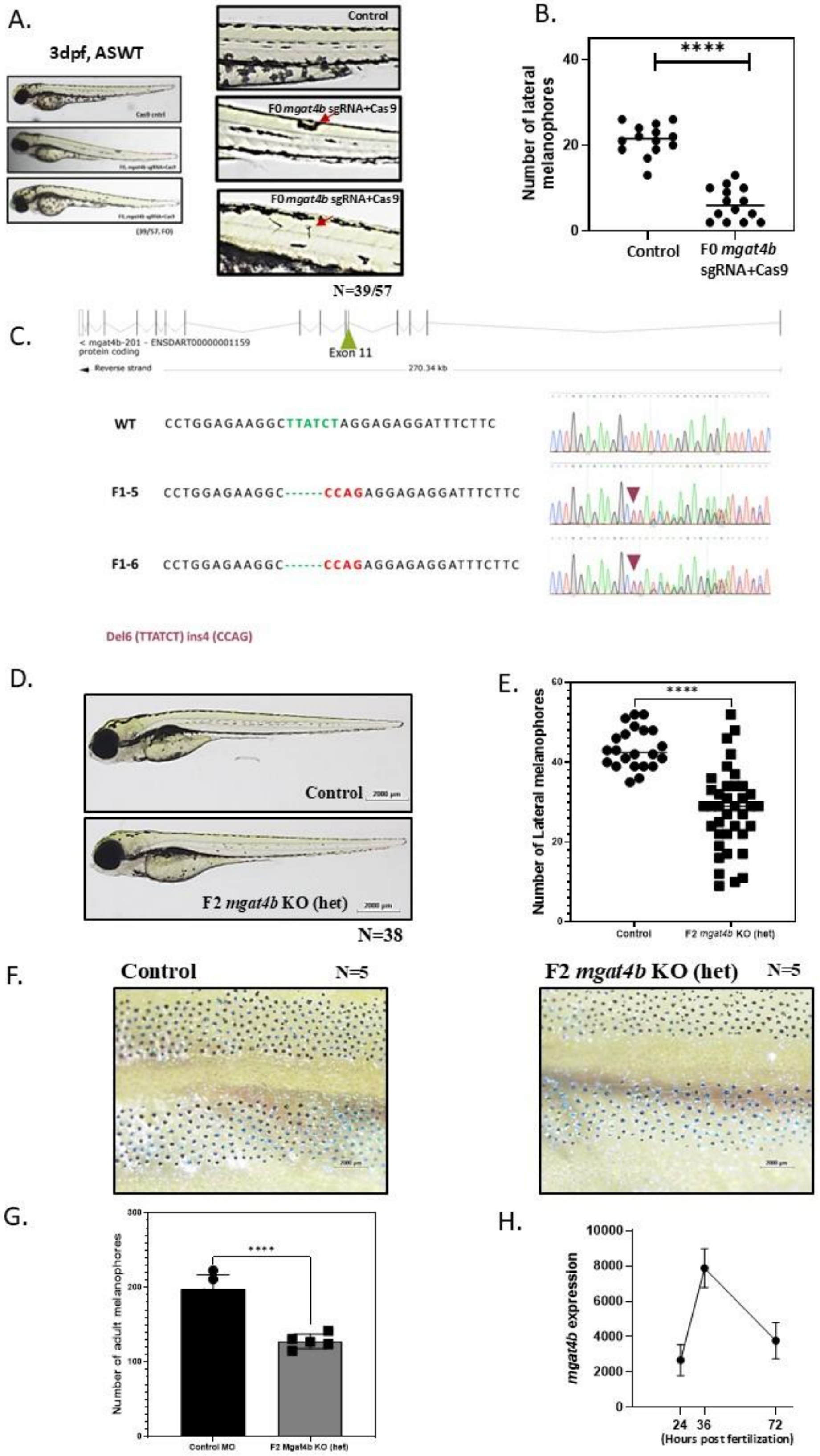
A.
3dpf, ASWT
Control
F0 mgat4b sgRNA+Cas9
F0 mgat4b sgRNA+Cas9
(39/57, F0)
N=39/57
B.
****
Number of lateral melanophores
Control
F0 mgat4b sgRNA+Cas9
C.
< mgat4b-201 - ENSDART00000001159
protein coding
Reverse strand
Exon 11
270.34 kb
WT CCTGGAGAAGGCTTATCTAGGAGAGGATTTCTTC
F1-5 CCTGGAGAAGGC------CCAGAGGAGAGGATTTCTTC
F1-6 CCTGGAGAAGGC------CCAGAGGAGAGGATTTCTTC
Del6 (TTATCT) ins4 (CCAG)
D.
Control
F2 mgat4b KO (het)
N=38
E.
****
Number of Lateral melanophores
Control
F2 mgat4b KO (het)
F.
Control N=5
F2 mgat4b KO (het) N=5
G.
****
Number of adult melanophores
Control MO
F2 Mgat4b KO (het)
H.
mgat4b expression
24 36 72
(Hours post fertilization)

Supplementary Figure 2 Global ablation of *mgat4b* shows patterning defects A. Brightfield images of F0 cas9 control and *mgat4b* sgRNA+Cas9 animals at 3dpf stage (left). Zoomed-in insets show black dendritic cells arrested along the dorso-lateral path in *mgat4b* sgRNA+Cas9 animals, which are absent in Cas9 controls (right) Scale bar 100 µm, Red arrows highlights the arrested cells **B.** Scatter plot depicting the number of lateral melanophores at 3 dpf stage of F0 cas9 control and *mgat4b* sgRNA+Cas9 animals **C.** Validation of F1 *mgat4b* knockout animals by sanger sequencing **D.** Brightfield images of F2 control and *mgat4b KO* F1xF1 animals at 4dpf stage (N=38) **E.** Scatter plot representing number of lateral melanophores in F2 control and *mgat4b KO* F1xF1 animals at 4dpf stage **F.** Brightfield images of adult High Resolution Melting Analysis (HRMA) validated F2 control and *mgat4b KO* F1xF1 animals shows constricted melanophores upon epinephrine treatment, Scale bar: 2000 µm **G.** Bar plot depicting number of melanophores in adult stripes across HRMA validated F2 control and *mgat4b KO* F1xF1 animals (N=5 each) **H.** Dot plot depicting the RNA levels of *mgat4b* in melanophores at three different stages of their development (24, 36 and 72 hours post fertilization). Re-analysed Zebrafish time-course microarray data (GSE189059)

*p ≤ 0.05, **p ≤ 0.01, ***p ≤ 0.001, ****p ≤ 0.0001 and ns p > 0.05

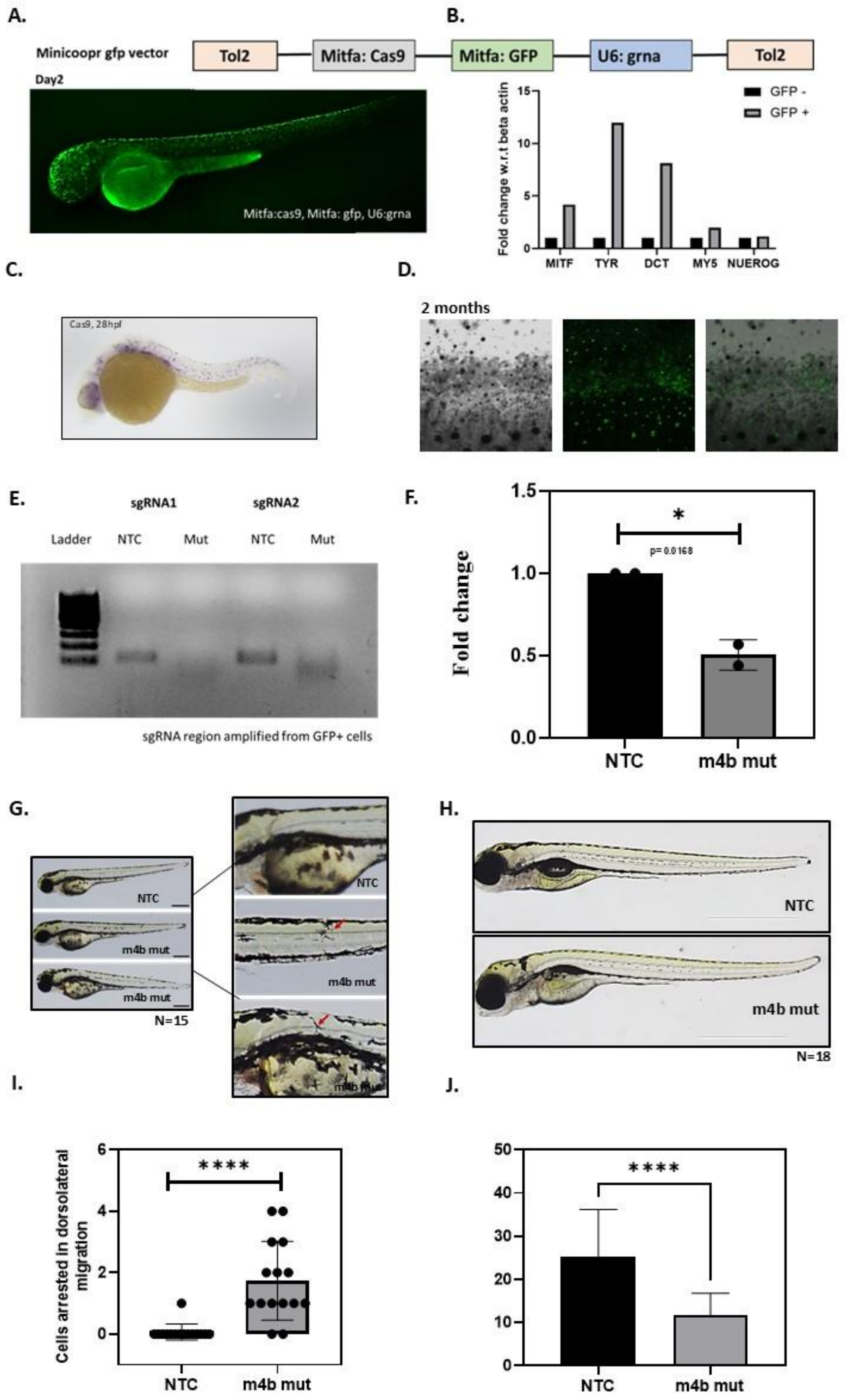
A.
B.
Minicoopr gfp vector
Tol2
Mitfa: Cas9
Mitfa: GFP
U6: grna
Tol2
Day2
Mitfa:cas9, Mitfa: gfp, U6:grna
Fold change w.r.t beta actin
GFP -
GFP +
MITF
TYR
DCT
MY5
NUEROG
C.
D.
Cas9, 28hpf
2 months
E.
F.
sgRNA1
sgRNA2
Ladder
NTC
Mut
NTC
Mut
sgRNA region amplified from GFP+ cells
Fold change
p= 0.0168
NTC
m4b mut
G.
H.
NTC
m4b mut
m4b mut
N=15
NTC
m4b mut
N=18
I.
J.
Cells arrested in dorsolateral migration
NTC
m4b mut
NTC
m4b mut

Supplementary Figure 3 Melanocyte-specific ablation of genes and visualization in zebrafish

A. Fluorescent image of 2dpf injected with *mitfa*:Cas9;*mitfa*:gfp plasmid at a single cell stage **B.** The bar plot presents transcriptomic levels of melanocyte-specific genes *mitf*, *tyr*, and *dct* in FACS-sorted GFP+ cells, alongside muscle and neuron-related negative controls **C.** WISH shows Cas9 expression in 28hpf zebrafish injected with *mitfa*:cas9;*mitfa*:gfp plasmid at a single cell stage **D.** Confocal images of 2 month old zebrafish injected with *mitfa*:cas9;*mitfa*:gfp plasmid at a single cell stage shows gfp+ cells **E.** T7 endonuclease I based melanocyte-specific *mgat4b* mutation detection. Agarose gel image shows PCR amplicons specific to sgRNA1 and sgRNA4 target sites from melanophores (GFP+ cells) of NTC and *mgat4b* mutant animals after cleavage **F.** qRT-PCR for *mgat4b* transcripts in FACS sorted gfp+ cells upon tissue-specific targeting of *mgat4b* in melanophores. Fold change is depicted (mean ± SEM, $n \geq 2$, biological replicates) calculated using beta actin as the reference with respect to non-targeting control animals **G.** Brightfield lateral images of m4b mut and NTC animals at 3dpf(left) Zoomed images highlighting cells arrested in the dorsolateral migration, pointed by red arrows for better clarity **H.** Brightfield lateral images depicting melanophore stripes of m4b mut and NTC animal at 7 dpf **H.** Bar plot depicting the number of melanophores arrested in between the path for m4b mut and NTC animals, N=15 **I.** Bar plot depicting the number of lateral melanophores in m4b mut and NTC animals, N=18

$^{*}p \leq 0.05$, $^{**}p \leq 0.01$, $^{***}p \leq 0.001$, $^{****}p \leq 0.0001$ and ns $p > 0.05$

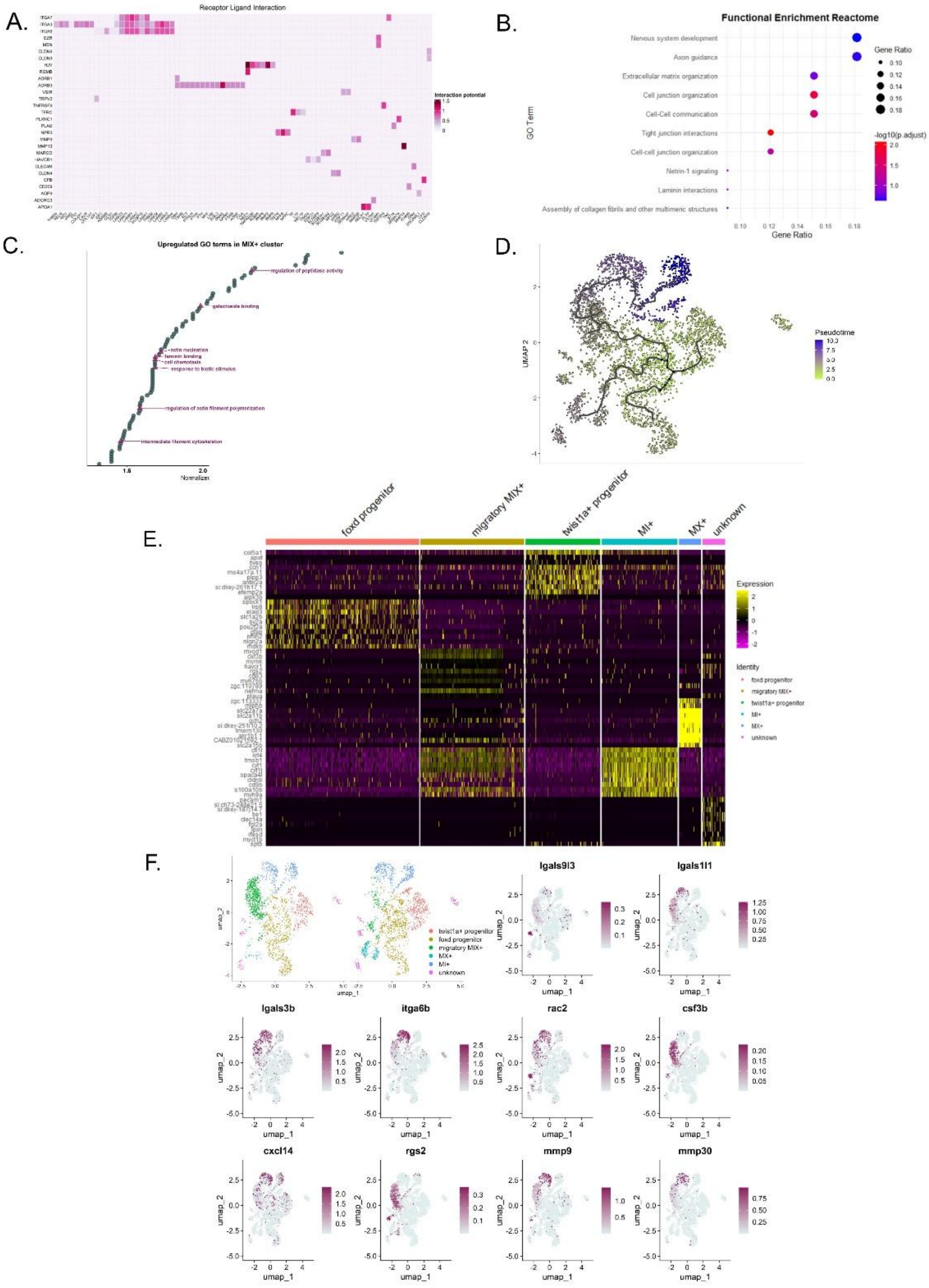
A.
Receptor Ligand Interaction
Interaction potential
B.
Functional Enrichment Reactome
Nervous system development
Axon guidance
Extracellular matrix organization
Cell junction organization
Cell-Cell communication
Tight junction interactions
Cell-cell junction organization
Netrin-1 signaling
Laminin interactions
Assembly of collagen fibrils and other multimeric structures
GO Term
Gene Ratio
-log10(p.adjust)
C.
Upregulated GO terms in MIX+ cluster
regulation of peptidase activity
galactoside binding
actin nucleation
laminin binding
cell chemotaxis
response to biotic stimulus
regulation of actin filament polymerization
intermediate filament cytoskeleton
D.
Pseudotime
UMAP 2
E.
foxd progenitor
migratory MIX+
twist1a+ progenitor
MI+
MX+
unknown
Expression
Identity
F.
lgals9l3
lgals1l1
lgals3b
itga6b
rac2
csf3b
cxcl14
rgs2
mmp9
mmp30
umap_1
umap_2

Supplementary Figure 4 scRNA seq reveals the identity of the depleted melanocyte population

A. Heatmap of NicheNetR-identified ligand/receptor pairs indicating interaction potential between MIX+cluster cells and other sender cells sampled by scRNAseq **B.** Functional enrichment for reactome of top enriched marker genes of MIX+ cluster **C.** Waterfall plot depicting top upregulated GO-terms in MIX+ cluster plotted against their normalized enrichment score **D**. Pseudotime ordering of the cells, coloring based on pseudotime scores **E.** Heat map depicting top markers enriched in each cluster and used to annotate them **F.** UMAPs of *mitfa*+ cells in NTC and *mgat4b* mut with color change from gray (negative) to purple based on log normalized scaled expression of *lgals9l3, lgals91l1, lgals3b, itga6b, rac2, csf3b, cxcl14, rgs2, mmp9, mm30* genes

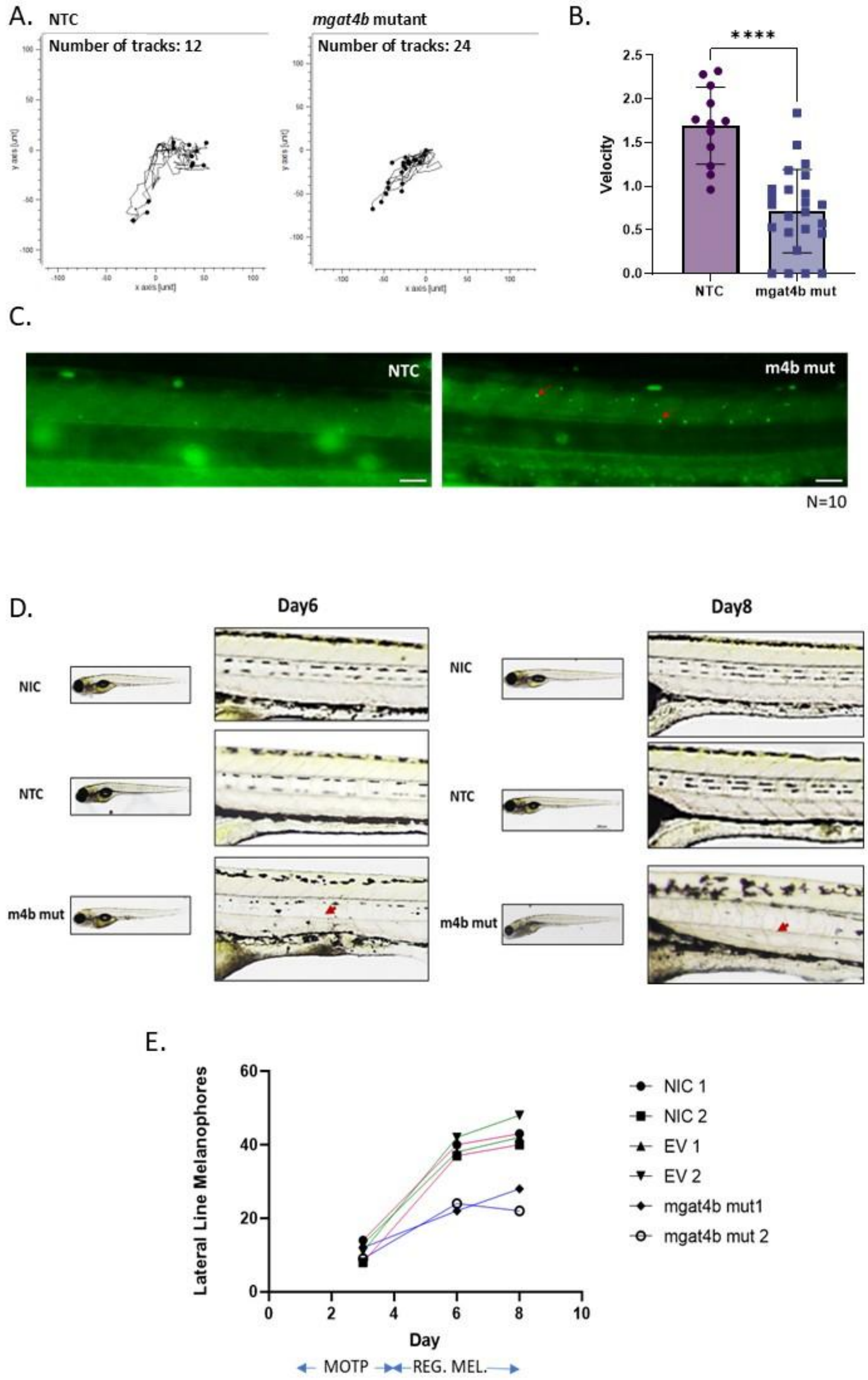
A.
NTC
Number of tracks: 12
mgat4b mutant
Number of tracks: 24
y axis [unit]
x axis [unit]
B.
****
Velocity
NTC
mgat4b mut
C.
NTC
m4b mut
N=10
D.
Day6
Day8
NIC
NTC
m4b mut
E.
Lateral Line Melanophores
Day
NIC 1
NIC 2
EV 1
EV 2
mgat4b mut1
mgat4b mut 2
MOTP
REG. MEL.

Supplementary Figure 5: Targeting *mgat4b* in melanophores affects their migration and regeneration capacity

A. Directionality plots depicting relative positioning of the cells in 12 hours timelapse imaging, compared to the start of the imaging is plotted as a 2D plot using chemotaxis and migration tool by Ibidi. NTC (left) *mgat4b* mutant (right) **B.** The bar plot illustrates the velocities of traced melanophores during migration in time-lapse imaging videos. Velocity was calculated based on the net movement in the 12h of imaging. The relative X and Y coordinates of the migrating cells was traced using Manual tracking plugin of Image J software **C.** Lateral images of 2dpf NTC and *mgat4b* mutant animal showing acridine orange puncta. Red arrows show puncta in the region where melanophore residing regions, Scale bars: 50 µm, N=10 each **D.** Brightfield images illustrate a melanocyte regeneration experiment in embryos subjected to MoTP-based ablation from Day 1 to Day 4, followed by regeneration from Day 4 to Day 8. Regenerating pigmented melanophores in the trunk region (lateral melanophores) were analyzed at Day 6 and Day 8 in non-injected controls, non-targeting controls, and *mgat4b* mutants **E.** The accompanying graph presents larval melanophore counts in non-injected controls (green lines), non-targeting controls (red lines), and *mgat4b* mutants (blue lines). Following the emergence of melanocytes at 24 hours post-MoTP treatment over three consecutive days, melanocyte numbers decline until Day 4, after which melanophores regenerate, leading to a subsequent increase in their numbers from Day 6 to Day 8

*$p \leq 0.05$, **$p \leq 0.01$, ***$p \leq 0.001$, ****$p \leq 0.0001$ and ns $p > 0.05$

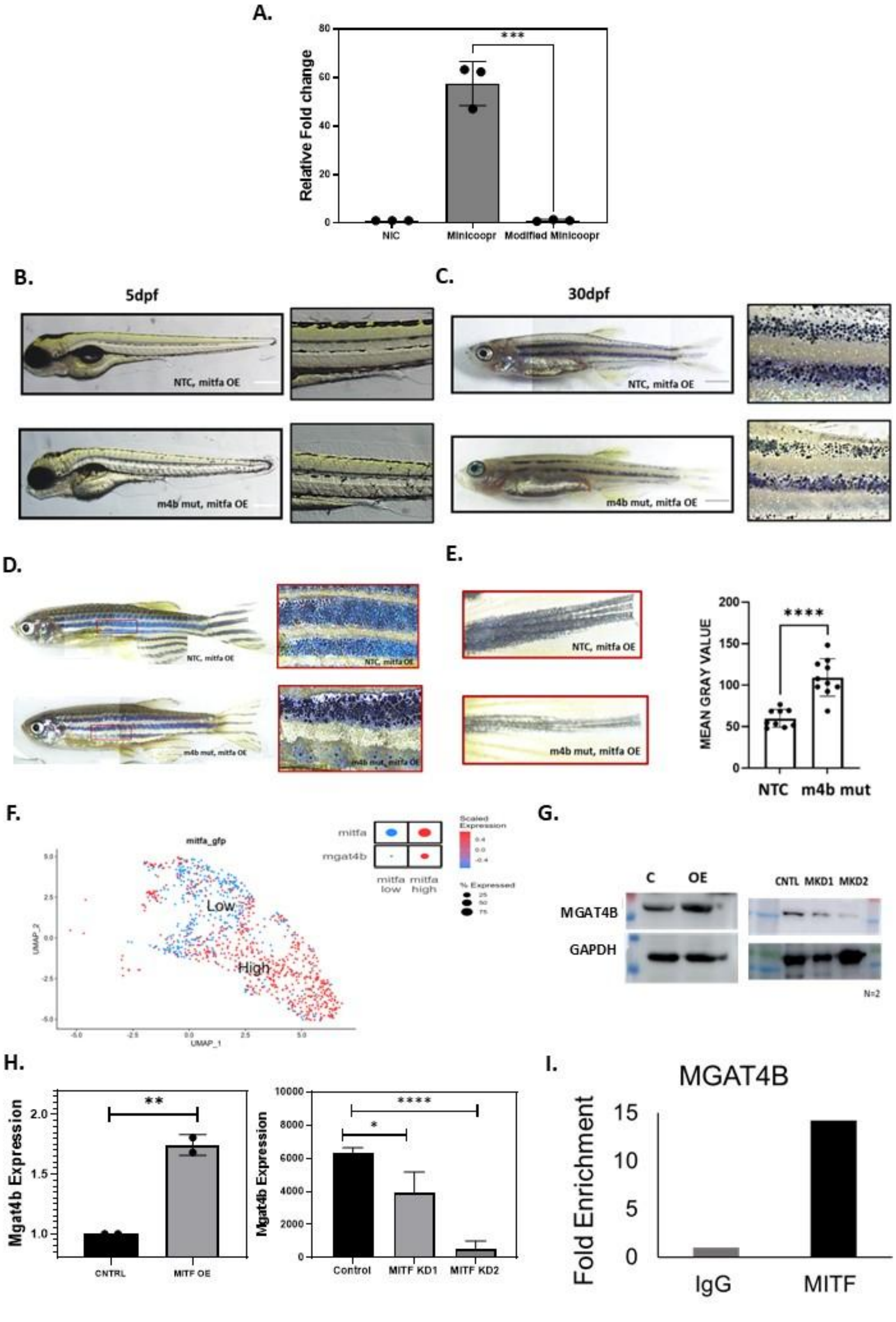
A.
Relative Fold change
***
NTC
Minicoopr
Modified Minicoopr
B.
5dpf
NTC, mitfa OE
m4b mut, mitfa OE
C.
30dpf
NTC, mitfa OE
m4b mut, mitfa OE
D.
E.
NTC, mitfa OE
m4b mut, mitfa OE
MEAN GRAY VALUE
****
NTC
m4b mut
F.
mitfa_gfp
Low
High
UMAP_1
UMAP_2
mitfa
mgat4b
mitfa low
mitfa high
Scaled Expression
% Expressed
G.
C
OE
CNTL
MKD1
MKD2
MGAT4B
GAPDH
N=2
H.
Mgat4b Expression
**
CNTRL
MITF OE
*
****
Control
MITF KD1
MITF KD2
I.
MGAT4B
Fold Enrichment
IgG
MITF

Supplementary Figure 6: **Central transcription factor MITFA transcriptionally regulates levels of MGAT4B in melanocytes: A.** The bar plot illustrates the relative fold change in *mitfa* transcripts in zebrafish clutches injected with the MinicoopR vector, modified MinicoopR vector, and non-injected controls **B.** Lateral images of 5dpf NTC and melanocyte-specific *mgat4b* mutant (m4b mut) zebrafish, showing embryonic melanocytic stripes with concurrent mitfa expression specifically in melanophores **C.** Lateral images of 30dpf NTC and *mgat4b* mut animals showing adult stripe patterns, zoomed images shows pigment stripes in the trunk region **D.** Lateral images of 50dpf NTC and *mgat4b* mut animals showing adult stripe patterns, zoomed images shows piment stripes in the trunk region **E**. Zoomed images of tail fin from 50dpf NTC and *mgat4b* mut animals, Bar graph represents mean±s.e.m. of mean gray value of tail fin selected ROI in NTC and *mgat4b* mut, each dot represents an animal **F**. UMAP plot showing *mitfa* high (red) and *mitfa* low(blue) cell clusters, dot plot showing collective expression of *mitfa* and *mgat4b* in these cells **G.** Western blot showing levels of MGAT4B upon overexpression of MITF in B16 mouse melanoma cells **,** Western blot showing expression of MGAT4B upon downregulation of MITF using two different siRNAs **H)** Bar graph showing quantitation of G **I.** Bar plot showing fold enrichment of MITF over MGAT4B promotor region revealed by ChIP-qPCR

*p ≤ 0.05, **p ≤ 0.01, ***p ≤ 0.001, ****p ≤ 0.0001 and ns p > 0.05

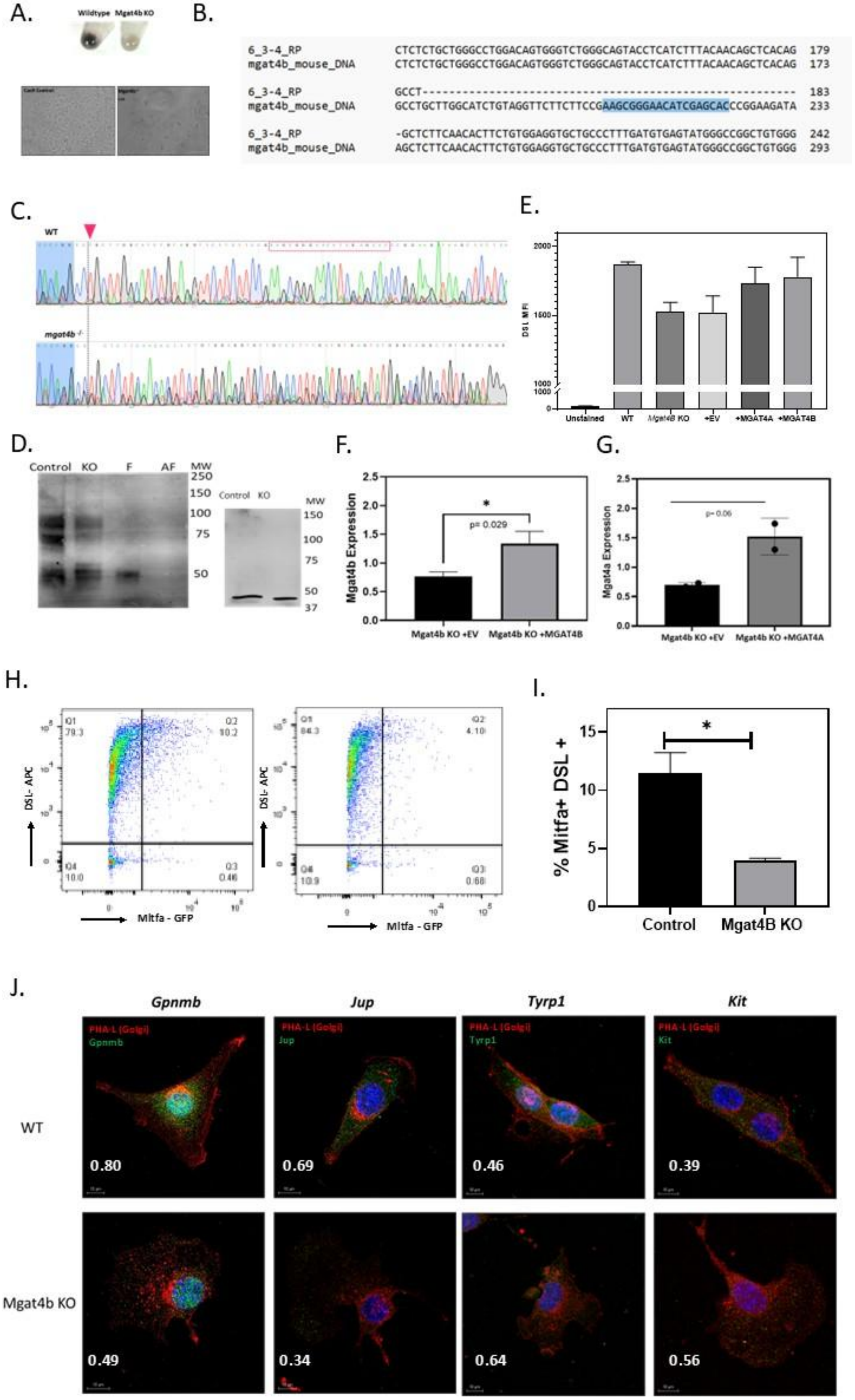
A.
Wildtype
Mgat4b KO
B.
6_3-4_RP CTCTCTGCTGGGCCTGGACAGTGGGTCTGGGCAGTACCTCATCTTTACAACAGCTCACAG 179
mgat4b_mouse_DNA CTCTCTGCTGGGCCTGGACAGTGGGTCTGGGCAGTACCTCATCTTTACAACAGCTCACAG 173
6_3-4_RP GCCT--- 183
mgat4b_mouse_DNA GCCTGCTTGGCATCTGTAGGTTCTTCTTCCGAAGCGGGAACATCGAGCACCCGGAAGATA 233
6_3-4_RP -GCTCTTCAACACTTCTGTGGAGGTGCTGCCCTTTGATGTGAGTATGGGCCGGCTGTGGG 242
mgat4b_mouse_DNA AGCTCTTCAACACTTCTGTGGAGGTGCTGCCCTTTGATGTGAGTATGGGCCGGCTGTGGG 293
C.
WT
mgat4b -/-
E.
DSL MFI
2000
1500
1000
0
Unstained
WT
Mgat4B KO
+EV
+MGAT4A
+MGAT4B
D.
Control
KO
F
AF
MW
250
150
100
75
50
Control
KO
MW
150
100
75
50
37
F.
Mgat4b Expression
2.5
2.0
1.5
1.0
0.5
0.0
*
p= 0.029
Mgat4b KO +EV
Mgat4b KO +MGAT4B
G.
Mgat4a Expression
2.5
2.0
1.5
1.0
0.5
0.0
p= 0.06
Mgat4b KO +EV
Mgat4b KO +MGAT4A
H.
Q1
79.3
Q2
10.2
Q4
10.0
Q3
0.46
DSL- APC
Mitfa - GFP
Q1
84.3
Q2
4.10
Q4
10.9
Q3
0.68
DSL- APC
Mitfa - GFP
I.
% Mitfa+ DSL +
15
10
5
0
*
Control
Mgat4B KO
J.
Gpnmb
Jup
Tyrp1
Kit
PHA-L (Golgi)
Gpnmb
PHA-L (Golgi)
Jup
PHA-L (Golgi)
Tyrp1
PHA-L (Golgi)
Kit
WT
0.80
0.69
0.46
0.39
Mgat4b KO
0.49
0.34
0.64
0.56

Supplementary Figure 7: ***Mgat4b* knockout leads to change in melanoma cells properties. A.** Cell pellet of day 7 B16 mouse melanoma wildtype and *Mgat4b* KO cells grown at low density (100 cells/cm2, top) brightfield images highlight the 'loose aggregate' phenotype in *Mgat4b* knockout embryos compared to wild-type, which exhibit well-aggregated colonies (bottom) **B.** Sanger sequencing based validation of *Mgat4b* knockout (colony 6, Mut1) in B16 mouse melanoma cells compared with wildtype sequence **C.** Sanger sequencing chromatogram of wild type (WT) and *Mgat4b* knockout (*Mgat4b*$^{-/-}$) clone shows deletion of 57 bp around the highlighted sgRNA region, The red arrowhead indicates the cleavage point in the knockout, compared to the wild-type **D.** DSL lectin blot depicting enrichment in wildtype and *Mgat4b* KO whole cell lysate with positive control (Fetuin) and negative control (asialofetuin) in place, beta-actin levels assessed as a loading control **E.** Bar plot shows mean±s.e.m of DSL lectin enrichment on the cell surfaces of Wildtype, *Mgat4b* KO, *Mgat4b* KO cells complemented with Empty vector, *Mgat4b* and *Mgat4a* constructs **F, G.** *Mgat4b* and *Mgat4a* expression in *Mgat4b* KO cells upon complementation of *Mgat4b* and *Mgat4a* construct, fold change calculated with respect to B16 WT cells **H.** FACS plot shows enrichment of DSL lectin on the cell surfaces of zebrafish NTC and *mgat4b* mutant cells **I.** Bar plot depicting the frequency of double positive cells (GFP+, DSL+) in NTC and *mgat4b* mutant melanocytes **J.** Confocal images of immunocytochemistry performed using *Tyrp1*, *Kit*, *Gpnmb* and *Jup* antibodies (green) along with PHA-L (Phaseolus Vulgaris Leucoagglutinin lectin (golgi marker, red)) on permeabilized wildtype and *Mgat4b* KO cells, counterstained by DAPI(blue). The overlay of all three channels is depicted. Mander's colocalization coefficient is displayed in white at the bottom left corner of each cell image, Scale bars: 10 µm

$^{*}p \leq 0.05$, $^{**}p \leq 0.01$, $^{***}p \leq 0.001$, $^{****}p \leq 0.0001$ and ns $p > 0.05$

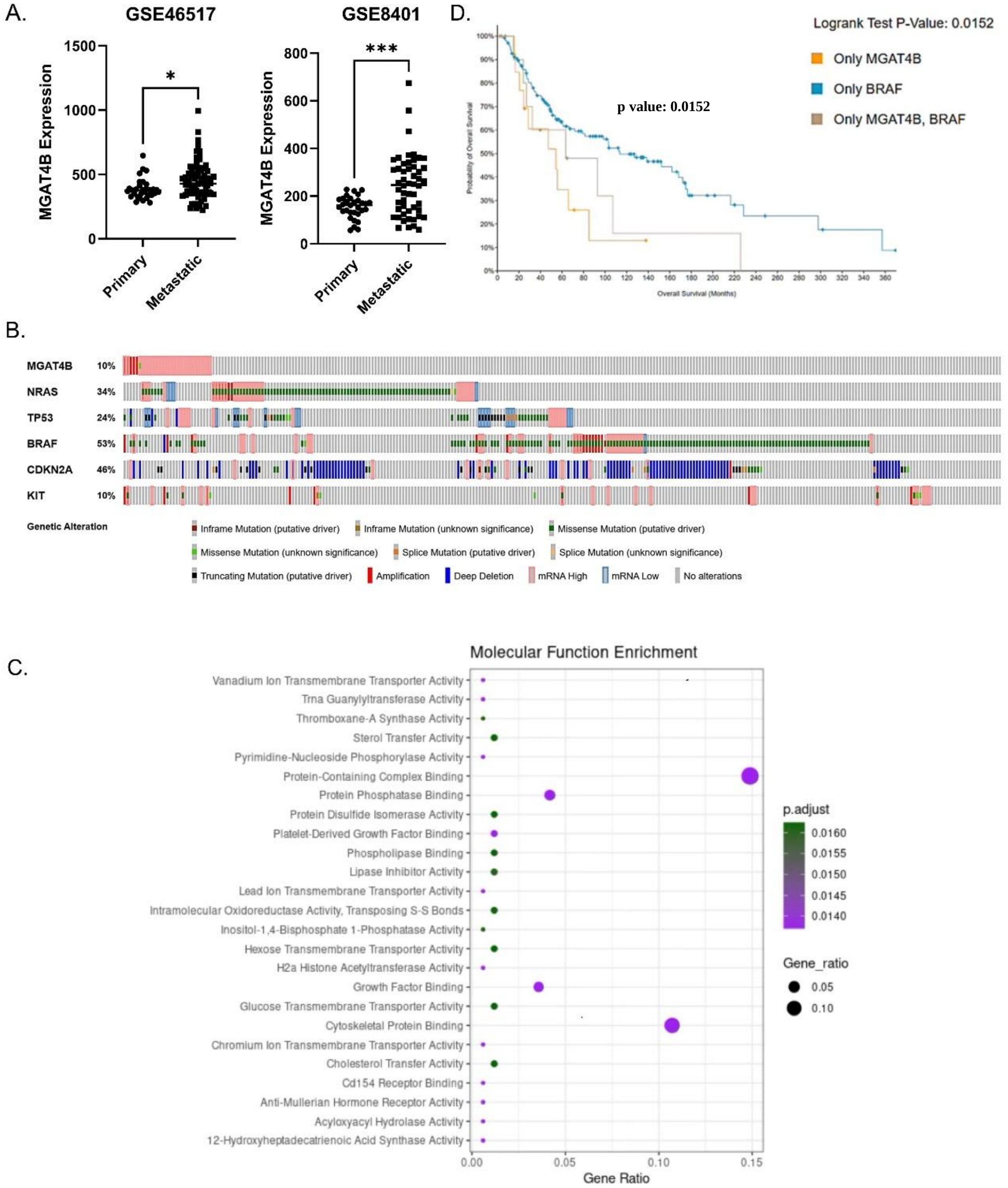

Supplementary figure 8: **Re-analysis of publicly available data shows enrichment of MGAT4B in melanoma patients**

A. Scatter plot depicts transcriptomic levels of MGAT4B in skin cutaneous melanoma patients segregated based on primary or metastatic stage, two different datasets were meta-analysed with the GSEA ID- GSE8401, GSE46517 **B.** Alteration frequency of candidate genes in the TCGA melanoma cohort, Top altered genes are plotted to display

deep deletions, missense mutations, and amplifications along with MGAT4B **C.** Molecular function enrichment of differentially regulated genes between MGAT4B high and MGAT4B low SKCM patients, data procured from TCGA, highlighted in red boxes are ECM and migration related terms **D.** Kaplan–Meier survival plots comparing overall survival (OS) probabilities (Y-axis) as a function of time in months (*x*-axis) in melanoma patients with mutated BRAF, MGAT4B and both BRAF and MGAT4B, Logrank test p value 0.0152

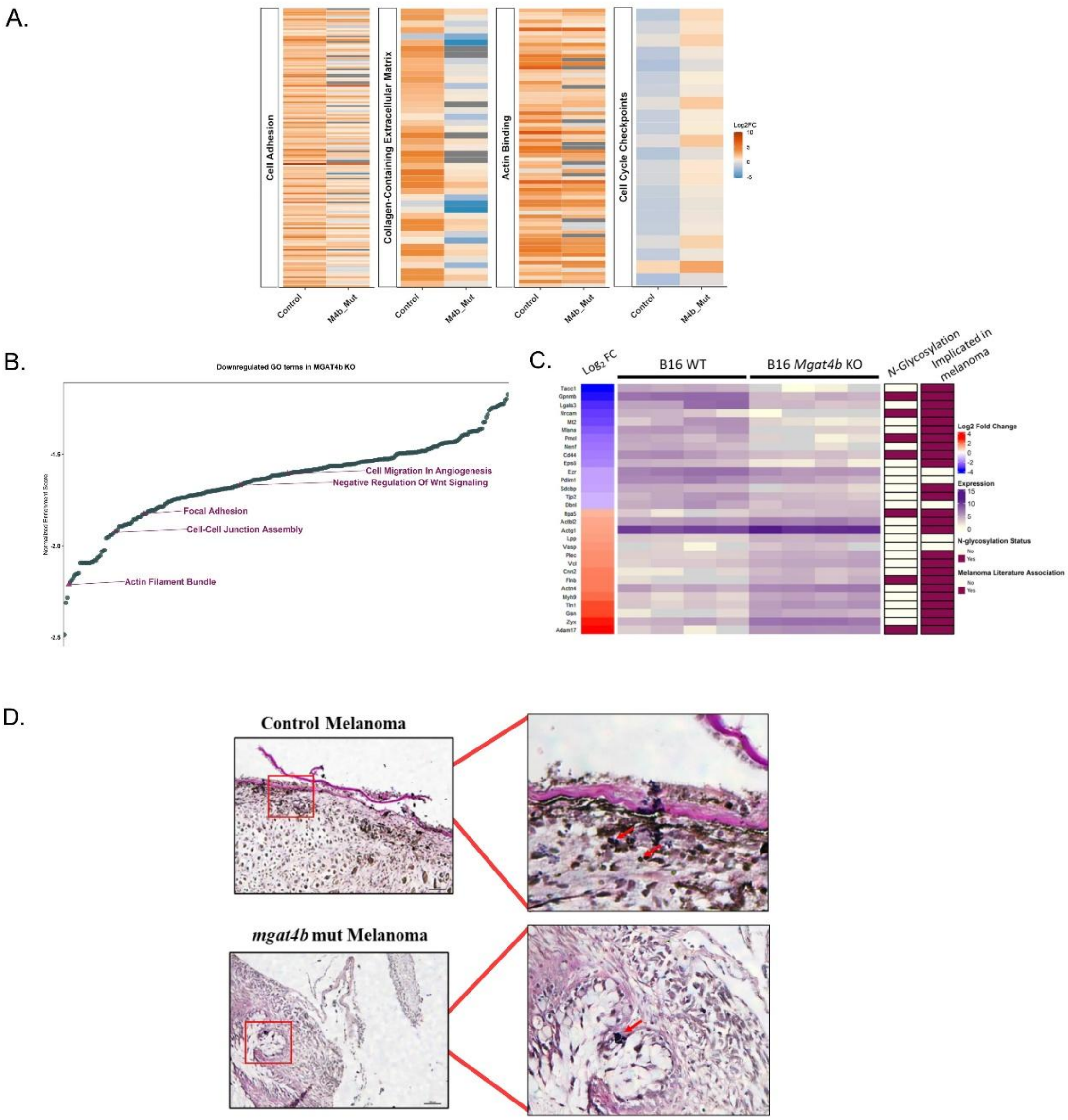

Supplementary figure 9: **Altered Cell Adhesion in zebrafish *mgat4b* mutants and B16 KO Cells**:

A. Heap map depicting log2 fold change of genes related to the GO terms cell adhesion, collage-containing extracellular matrix, actin binding and cell cycle check points in control melanoma (control), *mgat4b* mutant (M4b_Mut) melanoma with respect to wildtype melanophores **B.** Waterfall plot depicting normalized enrichment score of downregulated

GO terms in *Mgat4b* KO with respect to wildtype cells (B16 mouse melanoma), keys terms are highlighted **C.** Heap map depicting $\log_2$ fold change, $\log_2$ expression, *N*-glycosylation status and melanoma association of top differentially regulated proteins from B16 WT and B16 *Mgat4b* KO cells **D.** Visualization of collagen fibers near the tumor in both wild-type and *mgat4b* mutant melanoma in zebrafish. Verhoeff-Van Gieson (VVG) staining was performed on melanoma sections from both samples. Since the mutation is melanocyte-specific, the changes are not pronounced, as the majority of skin collagen is secreted by fibroblasts. Consequently, no significant differences in collagen staining around tumor cells were observed. Red insets highlight zoomed-in areas surrounding black melanoma cells in wild-type (top) and *mgat4b* mutant (bottom)

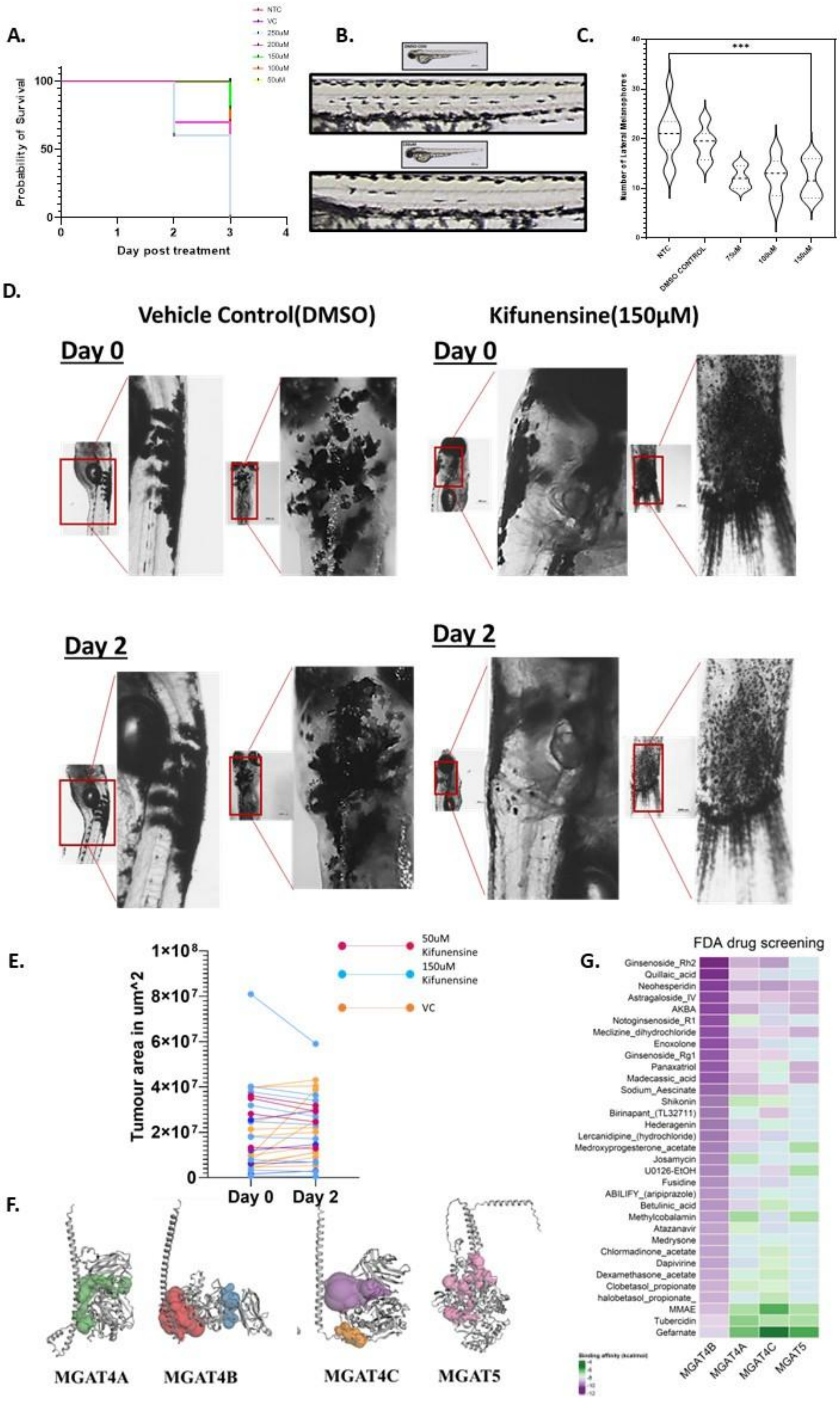
A.
NTC
VC
250uM
200uM
150uM
100uM
50uM
Probability of Survival
Day post treatment
B.
C.
Number of Lateral Melanophores
***
NTC
DMSO CONTROL
75uM
100uM
150uM
D.
Vehicle Control(DMSO)
Kifunensine(150μM)
Day 0
Day 0
Day 2
Day 2
E.
Tumour area in um^2
Day 0
Day 2
50uM Kifunensine
150uM Kifunensine
VC
F.
MGAT4A
MGAT4B
MGAT4C
MGAT5
G.
FDA drug screening
Ginsenoside_Rh2
Quillaic_acid
Neohesperidin
Astragaloside_IV
AKBA
Notoginsenoside_R1
Meclizine_dihydrochloride
Enoxolone
Ginsenoside_Rg1
Panaxatriol
Madecassic_acid
Sodium_Aescinate
Shikonin
Birinapant_(TL32711)
Hederagenin
Lercanidipine_(hydrochloride)
Medroxyprogesterone_acetate
Josamycin
U0126-EtOH
Fusidine
ABILIFY_(aripiprazole)
Betulinic_acid
Methylcobalamin
Atazanavir
Medrysone
Chlormadinone_acetate
Dapivirine
Dexamethasone_acetate
Clobetasol_propionate
halobetasol_propionate_
MMAE
Tubercidin
Gefarnate
MGAT4B
MGAT4A
MGAT4C
MGAT5

Supplementary Figure 10: Inhibition of complex *N*-glycosylation as a potential therapeutic intervention for melanoma initiation and progression

A. Survival curve showing mortality in early-developing ASWT zebrafish (3dpf) following treatment with varying concentrations of kifunensine (50, 100, 150, 200, and 250 μM) **B.** Brightfield images showing lateral melanophores in 3dpf ASWT fish treated with kifunensine (150 μM), with zoomed-in views of the trunk regions of each fish. **C.** Violin plot illustrating the number of lateral melanophores at 3dpf in zebrafish exposed to varying concentrations of kifunensine (50, 100, 150, 200, and 250 μM) **D.** One-month-old zebrafish with genetically initiated melanoma tumors, as previously described, were treated with the drug kifunensine. Grayscale images show the tumors at day 0, prior to treatment, and at day 2, after two days of drug treatment (150μM) in both the treated group and the vehicle control (DMSO) group **E.** Paired comparison graph depicts tumor area at day 0, prior to treatment, and at day 2, after two days of drug treatment (50 and 150μM) in both the treated group and the vehicle control (DMSO) group, Vehicle control (orange line), 50μM (red line) and 150μM (blue line) **F.** alpha-fold models of MGAT4A, MGAT4B, MGAT4C AND MGAT5 with their predicted binding pockets highlighted **G.** Heat map depicting the binding affinities of MGAT4A, MGAT4B, MGAT4C AND MGAT5 with the prioritised drugs.

$^*p \leq 0.05$, $^{**}p \leq 0.01$, $^{***}p \leq 0.001$, $^{****}p \leq 0.0001$ and ns $p > 0.05$

Datasets

Dataset S1: Cells velocity in the chemotaxis chamber when provided with a chemoattractant (SCF) added media or neutral media to control and B16 Wild type and B16 Mgat4b KO cells

Dataset S2: Proteomic analysis of whole cell lysates from wild type and Mgat4b KO B16 melanoma cells

Dataset S3: Proteomic analysis upon enrichment by DSL lectin in whole cell lysates of B16 Wild type and B16 Mgat4b KO cells

Dataset S4: Transcriptomic analysis of control and *mgat4b* mutant melanoma/melanophores from MAZERATI fishes with respect to wildtype melanophores from stage matched zebrafish. List of differentially expressed genes in NTC vs mgat4b mut melamnoma pertaining to collagen containing extracellular matrix

Dataset S5: List of markers utilized for annotating the specific clusters for the scRNA seq data. Comparison of cluster annotation markers with those identified in the scRNA-seq studies by Brombin et al. and Saunders et al.

Dataset S6: List of FDA approved drugs along with their predicted binding affinities to MGAT4A, MGAT4B, MGAT4C and MGAT5.

Supplementary Video 1: Time lapse imaging of 28 hpf zebrafish injected with *mitfa*:Cas9; *mitfa*:GFP: U6 sgRNA non-targetting plasmid as a control group

Supplementary Video 2: Time lapse imaging of 28 hpf zebrafish injected with *mitfa*:Cas9; *mitfa*:GFP: U6 *mgat4b* sgRNA plasmid as test group

Data Availability statement:

Transcriptomic data pertaining to single cell sequencing and RNA sequencing has been submitted to GEO repository
GSE278653 Mgat4b mediated selective *N*-glycosyl modification regulates melanocyte development and melanoma progression [bulk RNA-seq]
GSE278654 Mgat4b mediated selective *N*-glycosyl modification regulates melanocyte development and melanoma progression [scRNA-seq]

Proteomic data pertaining to Lectin Affinity enrichment and whole cell expression from B16 WT and B16 *Mgat4b* KO cells submitted to ProteomeXchange Consortium via the PRIDE partner repository
PXD056636 Proteomic data from B16 WT and B16 *Mgat4b* KO whole cell lysate
PXD056675 Proteomic data from Lectin affinity enrichment using DSL from B16 WT and B16 *Mgat4b* KO whole cell lysate

SI References

1. Zou, J., et al., *The Fugu tyrp1 promoter directs specific GFP expression in zebrafish: tools to study the RPE and the neural crest-derived melanophores.* Pigment cell research, 2006. **19**(6): p. 615-627.
2. Raja, D.A., et al., *pH-controlled histone acetylation amplifies melanocyte differentiation downstream of MITF.* EMBO reports, 2020. **21**(1): p. e48333.
3. Usui, Y., S. Kondo, and M. Watanabe, *Melanophore multinucleation pathways in zebrafish.* Development, Growth & Differentiation, 2018. **60**(7): p. 454-459.
4. Ablain, J., et al., *Human tumor genomics and zebrafish modeling identify SPRED1 loss as a driver of mucosal melanoma.* Science, 2018. **362**(6418): p. 1055-1060.
5. Liu, S., Z. Li, and J.-F. Gui, *Fish-specific duplicated dmrt2b contributes to a divergent function through Hedgehog pathway and maintains left-right asymmetry establishment function.* PLoS One, 2009. **4**(9): p. e7261.
6. Zheng, G.X., et al., *Massively parallel digital transcriptional profiling of single cells.* Nature communications, 2017. **8**(1): p. 14049.
7. Ihaka, R. and R. Gentleman, *R: a language for data analysis and graphics.* Journal of computational and graphical statistics, 1996. **5**(3): p. 299-314.
8. Satija, R., et al., *Spatial reconstruction of single-cell gene expression data.* Nature biotechnology, 2015. **33**(5): p. 495-502.
9. Ghojogh, B., et al., *Uniform manifold approximation and projection (UMAP)*, in *Elements of Dimensionality Reduction and Manifold Learning*. 2023, Springer. p. 479-497.
10. Yu, G., et al., *clusterProfiler: an R package for comparing biological themes among gene clusters.* Omics: a journal of integrative biology, 2012. **16**(5): p. 284-287.
11. Wickham, H., *ggplot2.* Wiley interdisciplinary reviews: computational statistics, 2011. **3**(2): p. 180-185.
12. Trapnell, C., et al., *The dynamics and regulators of cell fate decisions are revealed by pseudotemporal ordering of single cells.* Nature biotechnology, 2014. **32**(4): p. 381-386.
13. Cao, J., et al., *The single-cell transcriptional landscape of mammalian organogenesis.* Nature, 2019. **566**(7745): p. 496-502.
14. Qiu, X., et al., *Reversed graph embedding resolves complex single-cell trajectories.* Nature methods, 2017. **14**(10): p. 979-982.
15. Browaeys, R., W. Saelens, and Y. Saeys, *NicheNet: modeling intercellular communication by linking ligands to target genes.* Nature methods, 2020. **17**(2): p. 159-162.
16. Hu, Y., et al., *An integrative approach to ortholog prediction for disease-focused and other functional studies.* BMC bioinformatics, 2011. **12**: p. 1-16.
17. Higdon, C.W., R.D. Mitra, and S.L. Johnson, *Gene expression analysis of zebrafish melanocytes, iridophores, and retinal pigmented epithelium reveals*

indicators of biological function and developmental origin. PloS one, 2013. **8**(7): p. e67801.
18. Bolger, A.M., M. Lohse, and B. Usadel, *Trimmomatic: a flexible trimmer for Illumina sequence data.* Bioinformatics, 2014. **30**(15): p. 2114-2120.
19. Dobin, A., et al., *STAR: ultrafast universal RNA-seq aligner.* Bioinformatics, 2013. **29**(1): p. 15-21.
20. Liao, Y., G.K. Smyth, and W. Shi, *featureCounts: an efficient general purpose program for assigning sequence reads to genomic features.* Bioinformatics, 2014. **30**(7): p. 923-930.
21. Love, M., S. Anders, and W. Huber, *Differential analysis of count data–the DESeq2 package.* Genome Biol, 2014. **15**(550): p. 10-1186.
22. Sherman, B.T., et al., *DAVID: a web server for functional enrichment analysis and functional annotation of gene lists (2021 update).* Nucleic acids research, 2022. **50**(W1): p. W216-W221.
23. Upadhyay, S.K. and M. Papadakis. *Improving the visibility of underwater video in turbid aqueous environments*. in *SNAME Maritime Convention*. 2020. SNAME.
24. Da Silva, M. and M. Arruda, *Mechanization of the Bradford reaction for the spectrophotometric determination of total proteins.* Anal Biochem, 2006. **351**(1): p. 155-157.
25. Pv, A., et al., *Glycolytic state of aortic endothelium favors hematopoietic transition during the emergence of definitive hematopoiesis.* Science advances, 2024. **10**(7): p. eadh8478.
26. Varadi, M., et al., *AlphaFold Protein Structure Database: massively expanding the structural coverage of protein-sequence space with high-accuracy models.* Nucleic acids research, 2022. **50**(D1): p. D439-D444.
27. Trott, O. and A.J. Olson, *AutoDock Vina: improving the speed and accuracy of docking with a new scoring function, efficient optimization, and multithreading.* Journal of computational chemistry, 2010. **31**(2): p. 455-461.
